# An efficient brain delivery system co-loaded with multiple components of *Salvia miltiorrhiza* for synergistic treatment of ischemic stroke

**DOI:** 10.1016/j.mtbio.2025.102102

**Published:** 2025-07-16

**Authors:** Chenjie Xia, Changhui Hu, Rui Xu, Feihong Zhuo, Mengfei Yang, Yinjia Li, Zixuan Shan, Cheng Xu, Yutong Wang, Zhipeng Chen

**Affiliations:** a Key Laboratory of State Administration of TCM for Standardization of Chinese Medicine Processing, School of Pharmacy, Nanjing University of Chinese Medicine, Nanjing, 210023, China; b Department of Pharmacy, Wuxi Affiliated Hospital of Nanjing University of Chinese Medicine, Wuxi, 214071, China; c School of Biopharmacy, China Pharmaceutical University, Nanjing, 211198, China; d Pharmacy Department, Kunshan Hospital Affiliated to Nanjing University of Chinese Medicine, Suzhou, 215300, China

**Keywords:** *Salvia miltiorrhiza* Bge. (Danshen), Multi-component, Synergistic effect, Blood-brain barrier, Cerebral ischemic stroke

## Abstract

Ischemic stroke is a neurological disease characterized by high morbidity and mortality. A key pathological mechanism involves rapid restoration of blood oxygen in the infarcted area, which triggers oxidative stress and inflammatory responses leading to neuronal damage. The complex etiology of the disease poses challenges to single-target pharmacotherapy, necessitating the development of multi-targeted combination drug treatments. Reports showed that water- and lipid-soluble components of Danshen, exert synergistic neuroprotective effects. However, the disparate physicochemical properties of these compounds lead to differential release kinetics, hampering the therapeutic efficacy. Furthermore, the blood-brain barrier (BBB) presents a formidable obstacle to the penetration of water-soluble constituents. Here, we developed a novel biomimetic drug delivery system utilizing hollow polydopamine coated with red blood cell membranes. Water-soluble (W) and lipid-soluble (L) components of Danshen were loaded at distinct membrane regions to facilitate synchronized release profile. Additionally, borneol was used to modify the membrane to enhance the BBB penetration capacity. Mechanism study revealed that W and L possess synergistic effect through inhibiting NF-κB and TNF-α pathways. In summary, this delivery system enables concurrent loading and efficient brain delivery of multiple constituents, thereby promoting neurological recovery and presenting a promising therapeutic strategy for ischemic stroke.

## Key Highlights of our study include

• The co-delivery of water-soluble and lipid-soluble components achieves synergistic neuroprotection.
• The DDS exerts synchronized release kinetics, overcoming the limitations of differential release profiles.
• The incorporation of borneol facilitates reversible BBB opening, markedly improving the brain delivery efficiency.

## Graphical abstract

We developed an innovative brain-targeted biomimetic delivery system co-encapsulating five Danshen monomers for ischemic stroke therapy. Employing hollow polydopamine nanoparticles coated with red blood cell membranes and modified with borneol, we realized synchronized release and improved blood-brain barrier permeability, thereby presenting a fusion of nanotechnology and traditional Chinese medicine that significantly enhances therapeutic efficacy and patient prognosis, holding great potential for advancing stroke treatment.

**Figure:**
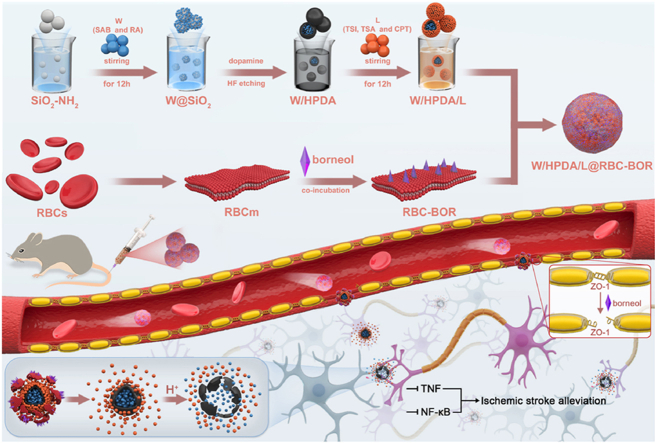

## 1 Introduction

The advent of an aging population has elevated cerebrovascular disease to a primary threat to human health and longevity. Ischemic stroke, in particular, presents a significant challenge due to its high incidence and mortality rates, imposing substantial social and economic burdens [1]. While timely and effective restoration of blood supply is crucial in its treatment, the process of recanalization paradoxically leads to severe cerebral ischemia-reperfusion injury, complicating clinical treatment and prognosis [2]. The onset of ischemic stroke triggers a cascade of secondary responses, including oxidative stress, inflammatory reactions, and cellular apoptosis. Among these, hyperactive inflammatory responses and excessive oxidative stress are the primary pathophysiological culprits responsible for neuronal damage induced by cerebral ischemia-reperfusion injury [[3], [4], [5]]. Therefore, how to protect neurons from oxidative stress and inflammatory response damage induced by cerebral ischemia-reperfusion is a critical issue that urgently needs to be addressed in the clinical treatment of ischemic stroke.

Given that neuronal damage caused by cerebral ischemia-reperfusion is a complex, cascading process involving multiple stages, a single-target therapeutic approach may not yield optimal results [6,7]. In this context, a multi-target combined treatment strategy presents a more viable solution. Traditional chinese medicine serves as an important natural resource repository for modern drug discovery, characterized by its multi-functional and multi-target properties [8]. Numerous active components from chinese herbal medicines have been demonstrated to exert potential therapeutic effects in ischemic stroke. *Salvia miltiorrhiza* Bge. (Danshen) has been widely employed in the treatment of various cardiovascular and cerebrovascular disorders [[9], [10], [11]]. The principal bioactive compounds in Danshen can be categorized into two groups: lipid-soluble tanshinones and water-soluble phenolic acids. Among the water-soluble active ingredients, rosmarinic acid (RA) and salvianolic acid B (SAB) are prominent, exhibiting potent antioxidant activities [12,13]. Studies have shown that RA can effectively scavenge reactive oxygen species (ROS) by enhancing endogenous antioxidant enzyme activity and reducing lipid peroxidation [14]. Jiang et al. discovered that SAB increased superoxide dismutase (SOD) activity and decreased malondialdehyde (MDA) levels in ischemic brain tissue of mice with cerebral ischemia and reperfusion injury, indicating SAB's neuroprotective properties [15]. Concurrently, lipid-soluble components in Danshen, such as tanshinone I (TSI), tanshinone IIA (TSA), and cryptotanshinone (CPT), have also been reported to possess neuroprotective effects. TSI has been reported to inhibit the activation of the NLRP3 inflammasome and protect against NLRP3-related diseases [16,17]. Research has shown that TSA exerts remarkable neuroprotective effects on ischemic stroke by modulating inflammatory cascades and neuronal signaling pathways involved in cerebral ischemia [18]. CPT has demonstrated therapeutic potential in ischemic stroke through enhancement of STAT5 phosphorylation [19]. However, current studies utilizing Danshen extract for stroke treatment often select only one component as the active substance, such as TSA [20,21]. These investigations neglect the potential synergistic effects among multiple components within the extract, thereby limiting therapeutic efficacy. Therefore, the co-delivery of these five distinct lipid-soluble or water-soluble active components derived from Danshen to the affected area for multi-target combined treatment of ischemic stroke presents a feasible approach to mitigate neuronal damage.

While co-administering five components of Danshen directly to the brain may potentially offer maximum efficacy, achieving synchronized release and optimal deposition control in the brain using traditional "cocktail" drug mixtures is challenging. This is due to the varying physicochemical properties (such as lipophilicity and hydrophilicity) and release behaviors of different drugs. In recent years, cascade release strategies have been widely employed to address the need for sequential drug delivery or release. Zhang et al. utilized mesoporous silica nanoparticles as the nanoconfined carrier to co-deliver doxorubicin and BMS777607, leading to synchronized and synergistic release of them for enhanced chemo-immunotherapy [22]. Drawing inspiration from this approach, we propose to adopt a cascade release strategy. This involves loading the lipophilic and hydrophilic components of Danshen into different compartments of the delivery system. By synchronizing the drug release behavior, we aim to achieve simultaneous release of drugs with different properties at the target site, thereby maximizing their synergistic therapeutic effects. Furthermore, to prevent premature drug release due to the clearance of the delivery system by the immune system during circulation, we have coated the delivery system with red blood cell (RBC) membranes. The CD47 protein on the RBC membrane can interact with signal regulatory protein-α, which inhibits the clearance of the erythrocyte membrane by the body's immune cells [23]. The microenvironment at the target site differs significantly from both the preparation conditions and systemic circulation. When the formulation reaches the acidic pathological environment, protonation occurs in the phospholipids of the outer RBC membrane [[24], [25], [26]]. This protonation disrupts the electrostatic balance of the lipid bilayer, potentially leading to either membrane fusion or rupture. Consequently, the RBC membrane detaches from the inner core nanoparticles, enabling drug release.

Another obstacle affecting the entry of Danshen compound components into the lesion site is the blood-brain barrier (BBB). The BBB is a highly selective semipermeable membrane barrier that exists between the brain's capillaries and brain tissue. This barrier is crucial for maintaining a stable environment and normal function of the brain [27,28]. However, the BBB also prevents most drugs, especially water-soluble components, from entering the brain, which hinders the accumulation of drugs at the lesion site in brain disease treatments, thereby affecting therapeutic efficacy [29,30]. Based on traditional Chinese medicine texts and theories, aromatic resuscitation drugs can serve as adjuvant agents targeting the brain, guiding active drugs to enter the brain more effectively [31]. Modifying drug delivery systems with aromatic resuscitation drugs can enhance the accumulation of active drugs in the brain, thereby improving therapeutic effects. For instance, it has been reported that borneol, an aromatic resuscitation drug, can easily penetrate the BBB, downregulate ZO-1 expression, and increase BBB permeability [32,33]. In the treatment of Alzheimer's disease, Jia et al. constructed a borneol-modified octahedral palladium (Pd@PEG@Bor) nanozyme platform. *In vitro* and *in vivo* experimental results demonstrated that Pd@PEG@Bor could cross the BBB and target neurons. Compared with the control group without borneol, the addition of borneol made it easier to penetrate the BBB and further ameliorate mitochondrial dysfunction [34]. Therefore, to promote greater penetration of the BBB by Danshen compound components and their entry into ischemic stroke lesion sites, we modified the red blood cell membrane [35,36] of the delivery system with borneol. This approach aims to achieve enhanced BBB penetration capability and increased drug accumulation at the lesion site.

**Scheme 1:**
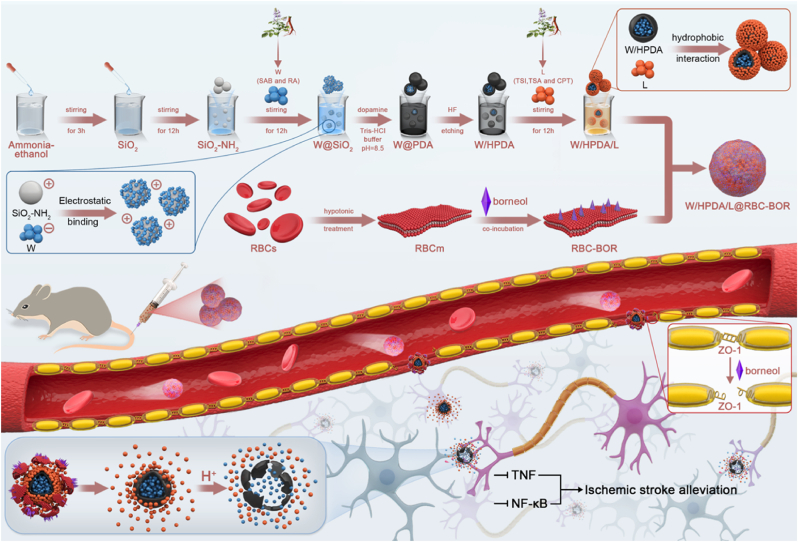
Schematic illustration of synthesis of W/HPDA/L@RBC-BOR nanosystem and the synergistic treatment of neuroinflammation in ischemia brain.

In the present study, we designed an efficient brain-targeted biomimetic delivery system co-loading five monomer components of Danshen (Scheme 1). Based on the different release behaviors of water-soluble and lipid-soluble components, active compounds (W and L) were confined in different spatial compartments. During the preparation of hollow polydopamine (HPDA), the silica template was loaded with W, ensuring their primary presence within the hollow polydopamine cavity. Subsequently, we utilized the surface groups of polydopamine nanoparticles to load L. This nano-confinement effect enables high-dose co-loading of different active compounds and, more importantly, leads to their synchronized release. Through the long-circulating properties of the RBC membrane and the BBB penetration mediated by borneol, the nanoparticles accumulate more effectively in the brain. In the acidic inflammatory microenvironment, the surface amino groups of HPDA undergo protonation [37,38], which subsequently alters the intermolecular interactions within HPDA. These changes may disrupt the balance of intermolecular forces in HPDA, triggering its degradation and ultimately leading to the release of the five active components from *Salvia miltiorrhiza*. This process enables their synergistic therapeutic effects, including anti-inflammatory action and reactive oxygen species scavenging. This study provides new insights for multi-modal treatment of stroke and promotes the clinical application of Danshen in ischemic stroke therapy.

## 2 Materials and methods

### 2.1 Materials

Rosmarinic acid(>98 %),Salvianolic acid B(>98 %),Cryptotanshinone(>98 %),Tanshinone I(>98 %),Tanshinone IIA(>98 %) and borneol(>98 %) were purchased from Shanghai Yuanye Biological Co.Ltd (Shanghai, China). Dopamine hydrochloride and tetraethyl orthosilicate (TEOS) were purchased from Aladdin Reagents (Shanghai, China). (3-aminopropyl) triethoxysilane (APTES) was acquired from *Macklin* Chemical Reagent Co., Ltd (Shanghai, China).

### 2.2 Cell culture

HT22, bEnd.3, and BV2 cell lines were cultured in DMEM supplemented with 10 % FBS, 100 IU/mL penicillin and 100 mg/mL streptomycin sulfate. The cell lines were cultured in a 5 % CO_2_ incubator at 37 °C. All cellular studies were performed by utilizing the cells at their logarithmic growth phase.

### 2.3 In vitro evaluation of the neuroprotective effects of Danshen extract and its active components

1 × 10^4^ cells/pore HT22 neuronal cells were seeded onto a 96-well plate and 3 × 10^5^ cells/pore HT22 neuronal cells were seeded onto a 6-well plate. Oxygen Glucose Deprivation/Reoxygenation (OGD/R) model was established as previously published [39]. After 2 h of OGD treatment, different compound or compounds combination (control, model, Danshen extract, W + L, RA, SAB, TSI, TSA, and CPT) were added into the medium, followed by 24 h incubation. Then the apoptosis rate of HT22 was measured with flow cytometry, and cell viability was detected with CCK-8 assay.

### 2.4 In vitro evaluation of the neuroprotective effects of Danshen components via different routes of administration

1 × 10^4^ cells/pore HT22 neuronal cells were seeded onto a 96-well plate and 3 × 10^5^ cells/pore HT22 neuronal cells were seeded onto a 6-well plate. OGD/R model was established as previously published [39]. After 2 h of OGD treatment, different compound or compound combination with different routes of administration (control, model, W, L, W-> L (sequential administration), and W + L (concurrent administration)) were added into the medium, followed by 24 h incubation. The experimental operation of W-> L group was to pre-administer W to HT22 cells for 24 h and then add L. Then the apoptosis rate of HT22 was measured with flow cytometry, and cell viability was detected with CCK-8 assay.

### 2.5 Preparation and characterization of W/HPDA/L@RBC-BOR nanoparticles

Preparation of W/HPDA/L nanoparticles: Briefly, 0.5 mL of TEOS were added over a stirred solution of 2 mL of NH_3_∙H_2_O solution in 100 mL of ethanol. The reaction mixture was vigorously stirred at room temperature for 3 h to obtain SiO_2_ nanoparticles. Then, 1.5 mL of APTES were dropped to the reaction mixture. Finally, SiO_2_-NH_2_ nanoparticles were collected by centrifugation at 10000 rpm for 10 min, then washed twice with ethanol and resuspended in ethanol for further use. As for the preparation of W@SiO_2_ nanoparticles, 1 mL of SiO_2_-NH_2_ nanoparticles and 4 mL of water components solution (1 mg RA and 12 mg SAB) were first mixed together. Under stirring for 12 h, the resultant W@SiO_2_ nanoparticles were acquired by centrifugation (10000 rpm, 10 min) and washed thrice with DI water. The total content of water-soluble in the supernatant (free RA and SAB) were determined by HPLC. W@SiO_2_ nanoparticles obtained by centrifugation were resuspended with Tris-HCl solution containing dopamine hydrochloride. PDA was prepared according to our precious report [40]. This was followed by the addition of 2 mL of etching solution. Then, through centrifugation (12000 rpm, 5 min) and washing with ultrapure water three times, W/HPDA nanoparticles were acquired for later use. After dissolving 6 mg of lipid-soluble components (1 mg CPT, 2.5 mg TSI and 2.5 mg TSA) in 4 mL of ethanol, it was added to 1 mL of W/HPDA nanoparticles dispersed solution. After 12 h of stirring at room temperature, W/HPDA/L nanoparticles were collected by centrifugation and subsequently dispersed in water for further use. Similarly, HPDA was synthesized as previously described. 13 mg of water-soluble components solution (1 mg RA and 12 mg SAB) and 6 mg of lipid-soluble components (1 mg CPT, 2.5 mg TSI and 2.5 mg TSA) were dissolved in 4 mL PBS containing 0.1 % DMSO and added to 3 mL HPDA aqueous dispersion. The mixture was then stirred at room temperature for 12 h, centrifuged, and washed to obtain HPDA/WL nanoparticles. The total content of lipid-soluble in the supernatant (free CPT, TSI and TSA) were determined by HPLC.

Preparation of RBC-membrane-derived vesicles and borneol-modified RBC membrane: Hypotonic hemolysis method was used for the preparation of RBC-membrane-derived vesicles as previously published [41]. The RBC-membrane-derived vesicles and borneol (1:1) were then dissolved with 10 mL of PBS containing 0.1 % DMSO and stirred overnight to obtain the RBC-BOR. The product was collected by centrifugation. The borneol in the supernatant was separated by adding water and analyzed by gas chromatography. For the preparation of W/HPDA/L@RBC-BOR nanoparticles, W/HPDA/L nanoparticles were mixed with borneol-modified RBC membrane vesicles and coextruded through 200 nm polycarbonate porous membranes for 3 times as previously published [42]. The size distribution and surface charge density of nanoparticles were determined by a Malvern Zetasizer Nano Instrument (ZS90, Malvern, UK). The size distribution and morphology of nanoparticles were further measured by transmission electron microscopy (TEM) (HT7800, Hitachi, Japan). Western blotting was conducted to assess the presence of specific protein markers CD47. The coating efficiency was quantitatively assessed using a fluorescence-based assay. Specifically, PDA core was fluorescently labeled, with its initial fluorescence intensity serving as a reference. Notably, an increase in the amount of cell membrane coating induces a corresponding decrease in fluorescence intensity. By calculating the percentage change in fluorescence intensity, the cell membrane coating efficiency can be quantitatively evaluated. Furthermore, the particle size of the W/HPDA/L@RBC-BOR was monitored continuously for 7 days to evaluate its storage stability.

### 2.6 Adsorption model fitting

An adsorption kinetic model was used to fit the effect of adsorption time on the adsorption performance of adsorbents, further speculating the mechanism of adsorption. The results of the adsorption kinetics of five components were fitted using the pseudo-first-order [43] and pseudo-second-order model [44], respectively.

### 2.7 In vitro drug release

*In vitro* cumulative release of HPDA/WL, W/HPDA/L and W/HPDA/L@RBC-BOR in 1 % SDS (pH 6.5–6.8) were studied. 1 mL of HPDA/WL, W/HPDA/L and W/HPDA/L@RBC-BOR was sealed in dialysis bags (3000 Da) and then submersed in 200 mL 1 % SDS (pH 6.5–6.8), shaken continuously at 37 °C. 5 mL sample was taken at each time point, which was replaced with 1 % SDS (pH 6.5–6.8). Five compounds were measured by HPLC at 0.5, 1, 2, 4, 8, 12 and 24 h. In order to elucidate the release mechanism of W/HPDA/L, release kinetic models (Ritger-Peppas model) were employed to fit the data available.

### 2.8 Evaluation of BBB permeability *in vitro*

6 × 10^4^ bEnd.3 cells were seeded in the donor chamber of 24-well transwell inserts. The BBB model was established when cell monolayers exhibited transendothelial electrical resistance (TEER) exceeding 200 Ω cm^2^ [45]. The viability of bEnd.3 cells following borneol treatment was assessed using the Cell Counting Kit-8 (CCK-8) assay. We added 0.9 mL of PBS-based assay medium to blank 24-well culture plates and 0.2 mL of borneol (0, 100, 400 μM) to the apical side of the BBB layers. TEER was measured at 5, 10, and 15 min. Additionally, 100 nm silica nanoparticles and sodium fluorescein at the same concentrations were administered at three time points. After 1 min, the basolateral chamber solution was collected to determine particle count and apparent permeability coefficient (Papp). The intracellular tension of the tight junction protein ZO-1 was evaluated using fluorescence resonance energy transfer (FRET)-based tension probes and cpstFRET analysis, as previously described [46]. bEnd.3 cells were transfected with ZO-1 probes and incubated with 1000 μg/mL geneticin. After treating the cells with borneol for 15 min, the borneol was removed, and observations continued for an additional 15 min. Cells treated with Hanks' solution served as the control group. The cell images were taken with × 63 oil lens on a confocal microscope (SP5; Leica, Wetzlar, Germany). The CFP/FRET ratio was calculated using the equation 1/E = cerulean donor/venus acceptor.

### 2.9 Cytotoxicity assays

CCK-8 assays were utilized to determine the cytotoxicity of HPDA@RBC-BOR and W/HPDA/L@RBC-BOR NPs. Simply, BV2 cells were seeded at 2 × 10^4^ per well and cultured in 96-well plates for 12 h. W/HPDA/L@RBC-BOR NPs were then diluted to final W concentrations of 1, 5, 10, 25 and 50 μg/mL in cell medium. HPDA@RBC-BOR NPs were based on the same concentration of W, and incubated for 24 h. HT22 and bEnd.3 cells were substituted for BV2 cells, and the cytotoxicity of NPs was determined without changing other steps.

### 2.10 In vitro evaluation of neuroprotective effects

OGD/R model was established as previously published [39]. HT22 neuronal cells (1 × 10^5^ cells per transwell, lower chamber) were cocultured with bEnd.3 cells (2 × 10^5^ cells per transwell, upper chamber) in 12-well plates of transwell system for 24 h. Prior to this, transwell culture inserts were placed into each well of 12-well plates. After 2 h of OGD treatment, different groups (control group, PBS group, free drugs group, W/HPDA/L group, W/HPDA/L@RBC group, W/HPDA@RBC-BOR group, HPDA/L@RBC-BOR group, W/HPDA/L@RBC-BOR group, and HPDA/WL@RBC-BOR group) of nanospheres were added into cell medium in the upper chamber for 12 h incubation. The depletion of the intracellular ROS in the cell model was monitored with a 2′,7′-Dichlorodihydrofluorescein diacetate (DCFH-DA) assay. Then intracellular DCFH fluorescence intensity was analyzed by flow cytometry. Cell apoptosis was detected by staining with annexin V-FITC and propidium iodide, and analyzed by flow cytometry. BV2 cells were substituted for HT22 cells, and the cells were treated without changing other steps. After 12 h, the cells in each treatment were digested with 0.25 % trypsin, then blown and collected in 1.5 mL PBS (4 °C) and centrifuged at 1500 rpm for 5 min, twice. Afterwards, the membrane surface molecules were stained with 3 μL/test of FITC-conjugated anti-mouse CD86 MAb or APC-conjugated anti-mouse CD206 MAb for 30 min at room temperature in the dark as per the manufacturer's instructions. The samples were washed thrice with PBS and suspended in 4 % paraformaldehyde. Mean fluorescence intensity (MFI) of the cell surface molecules was assessed by flow cytometry (FCM). At 12 h post-treatment, the cell medium at the down chamber was collected for ELISA tests performed by Mouse ELISA Kit of TNF-α, IL-1β and IL-10.

### 2.11 Pharmacokinetic study

*In vivo* pharmacokinetic testing of different formulations was carried out using SD rat. The rats were divided into different groups and intravenously injected with free drug, W/HPDA/L, W/HPDA/L@RBC and W/HPDA/L@RBC-BOR (equal to 2.11 mg/kg RA, 4.02 mg/kg SAB, 1.08 mg/kg CPT, 1.02 mg/kg TSI and 1.12 mg/kg TSA). At pre-established time points, blood samples were placed in a centrifuge tube containing heparin sodium for 0.5 h. After centrifuging at 6000 r/min for 15 min at 4 °C, the supernatant was separated and added methanol. The sample was injected into the UHPLC-MS/MS system for analysis. The animals were killed 12 h after administration, and the heart, liver, spleen, lung, kidney and brain tissues were collected. The drug concentration in each tissue was determined by sampling analysis.

### 2.12 The tissue distribution experiment

ICR mice (SPF, male, 25–30 g) were purchased from Bikewing Biotechnology Co. Ltd. (Shanghai, China). The Experimental Animal Management Committee of Nanjing University of Chinese Medicine (Nanjing, Jiangsu, China) approved all experimental procedures. Cy7.5 was separately packaged into three different nanoparticles, and finally obtained with fluorescence W/HPDA/L, W/HPDA/L@RBC and W/HPDA/L@RBC-BOR nanoparticles, which were injected into mouse tail vein. After 4, 8, 12 h of drug circulation, the mice were killed, and the body tissues: brain, heart, liver, spleen, lung and kidney were taken out and placed in the PerkinElmer IVIS Lumina III imaging system for detection.

### 2.13 In vivo anti-ischemic stroke efficacy

Male SD rats (240–250 g) were purchased from Bikewing Biotechnology Co. Ltd. (Shanghai, China). The procedures for the care of animals and all animal experiments were evaluated and approved by the Institutional Animal Care and Use Committee of Nanjing University of Chinese medicine. Rat Middle Cerebral Artery Occlusion/Reperfusion (MCAO/R) model was established with a reported monofilament method [47]. SD rats were weighed and randomly divided into eight groups. Sham-operated group was used as the negative control, and the model rats were intravenously injected with saline, free drugs, W/HPDA/L, W/HPDA/L@RBC, HPDA/L@RBC-BOR, W/HPDA@RBC-BOR, or W/HPDA/L@RBC-BOR nanoparticles (equal to 10.01 mg/kg RA, 19.98 mg/kg SAB, 4.97 mg/kg CPT, 5.02 mg/kg TSI and 4.96 mg/kg TSA). The neuroscore evaluation and infarct size measurement were conducted at 24 h after the MCAO/R surgery. Neuroscore assessment was performed using a well-established five-point scale methodology (Rating scale, 4 = spontaneous circling, 3 = circling to left by pulling the tail, 2 = decreased grip strength of left forepaw, 1 = failure to extent left forepaw, and 0 = no deficit). After the neuroscore assessment, the rats were sacrificed, and brain tissues were removed immediately and frozen at −20 °C for 5 min. The brain slices were made at 2 mm thick and then stained with 2 % 2,3,5-triphenyltetrazolium chloride (TTC) at 37 °C for 30 min. The TTC-stained sections were photographed, and the infarct area (white part) the images were quantified with ImageJ software.

The brain tissue fixed in 4 % paraformaldehyde for more than 24 h was removed, paraffin embedded, sliced and then hematoxylin and eosin (H&E), Nissl, neuronal nuclei (NeuN) and terminal deoxynucleotidyl transferase-mediated dUTP nick-end labeling (TUNEL) staining were performed. The pathological morphological changes, neuronal damage and apoptosis of brain tissue sections were observed under microscope, and the corresponding quantitative analysis was performed on NeuN and TUNEL staining sections.

### 2.14 ROS and inflammatory levels determination

The alleviation of oxidative stress in the ischemic brain hemisphere was evaluated by measuring the activities of oxidative stress indicators such as superoxide dismutase (SOD) and malondialdehyde (MDA) in the homogenate of the isolated ischemic brain. The inflammatory cytokines, tumor necrosis factor (TNF)-α, interleukin 6 (IL-6), and interleukin 1β (IL-1β), were also evaluated by ELISA kit.

### 2.15 Biosafety evaluation

On the day of treatment, as well as on days 7 and 21 post-treatment, the main organs, heart, liver, kidney, spleen and lung were taken from SD rats. After adequate fixation with 4 % paraformaldehyde. Paraffin embedding, section (4 μm), H&E staining, the histopathological changes after different treatments were observed. Blood samples were collected from each group for biochemical analysis. Liver and kidney function (ALT, ALP, AST and BUN) were measured by automatic biochemical analyzer.

### 2.16 Mechanism investigation

Total RNA was extracted using the TRIzol reagent (Invitrogen, CA, USA) following the manufacturer's protocol. RNA integrity was evaluated using an Agilent 2100 Bioanalyzer (Agilent Technologies, Santa Clara, CA, USA). The libraries were constructed using VAHTS Universal V6 RNA-seq Library Prep Kit according to the manufacturer's instructions and then sequenced on the Illumina Novaseq 6000 platform; 150-bp paired-end reads were generated. All sequencing processes and analyses were performed by Shanghai OE Biotech Co., Ltd. (Shanghai, China). The obtained results were imported into STROMICS database [48] to obtain the genes of cerebral ischemia disease. The expression of differential genes was evaluated using RT-qPCR. Relative gene expression was calculated using the 2-^△△CT^ method, with normalization to the Ct of the housekeeping gene (*GAPDH*). The primer sequences used are listed in Supplementary Table S1. The key proteins were verified by Western blot.

### 2.17 Statistical analysis

All data are presented as mean ± standard deviation (SD). One-way analysis of variance (ANOVA) and two-tailed Student's t-test were employed for data analysis.

## 3 Results and discussion

### 3.1 Neuroprotective equivalence of five-component mixture of Danshen blend to native extract and enhanced efficacy via synchronized dual-component release

**Fig. 1:**
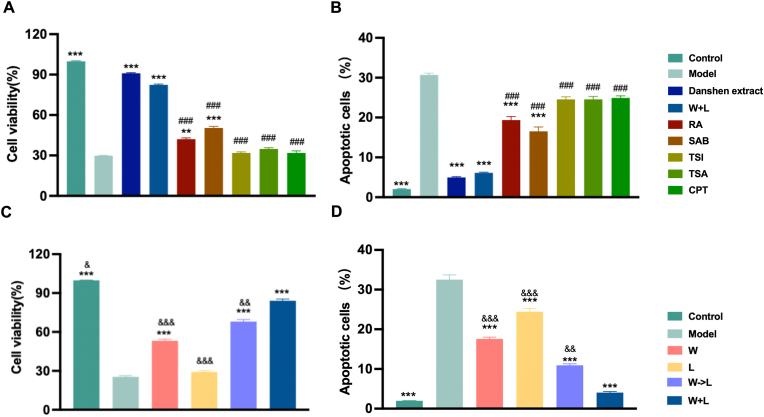
Comparative analysis of neuroprotective efficacy. Cell viability(A) and apoptosis(B) of HT22 cells incubated with different group (n = 6). Cell viability(C) and apoptosis(D) of HT22 cells incubated with different group (n = 6). Data are presented as mean ± SD. ∗∗*P* < 0.01, ∗∗∗*P* < 0.001 *vs.* model; ^###^*P* < 0.001 *vs.* Danshen extract group; ^&&^*P* < 0.01, ^&&&^*P* < 0.001 *vs.* W + L group.

To confirm the pharmacological equivalence between the five-component mixture of Danshen and its extract, we measured the viability and apoptosis rate of neuronal cells treated by different compound or compounds combination following oxygen-glucose deprivation/reoxygenation (OGD/R) injury. As illustrated in Fig. 1A, cell viability dropped to 29.91 ± 0.41 % following OGD/R exposure, confirming the successful establishment of the cellular model. Both *Danshen* extract and the five-component mixture significantly enhanced the cell viability compared to that of OGD/R group, with no statistically significant difference between the two groups. After OGD/R exposure, the apoptosis rate of HT22 cells increased significantly (30.71 ± 0.99 %), while Danshen extract and five compounds markedly reversed the above trend (Fig. 1B). Notably, individual monomeric constituents exhibited limited efficacy (Fig. 1B). These results demonstrated that the pharmacological effects of Danshen can be effectively replicated by a combination of five monomeric constituents, which exhibited a significant synergistic effect.

Next, we categorized the constituents of Dnashen into two groups—hydrophilic (W) and lipophilic (L) components—and compared their neuroprotective effects when administered simultaneously versus sequentially. As shown in Fig. 1C, both synchronous and asynchronous release strategies significantly enhanced cell viability (84.06 ± 3.37 % and 68.01 ± 4.01 %) compared to the model group, with the synchronous release group demonstrating superior efficacy (Fig. 1C), which was also validated by the cell apoptosis assay (Fig. 1D). These results demonstrate that the dual-component formulation outperforms single-class constituents, with synchronous release exhibiting superior therapeutic efficacy. While our main focus was to achieve synchronized release of both component types, we recognize that the pharmacological interactions between components at different ratios warrant more thorough investigation. Nevertheless, our pharmacodynamic comparisons did demonstrate that: the multi-component formulation exhibited significantly enhanced therapeutic efficacy compared to single-component treatments, with effects comparable to those of crude *Salvia* water extract. These results suggest no apparent antagonistic interactions between components at the tested ratio.

### 3.2 Preparation and characterization of W/HPDA/L nanoparticles

**Fig. 2:**
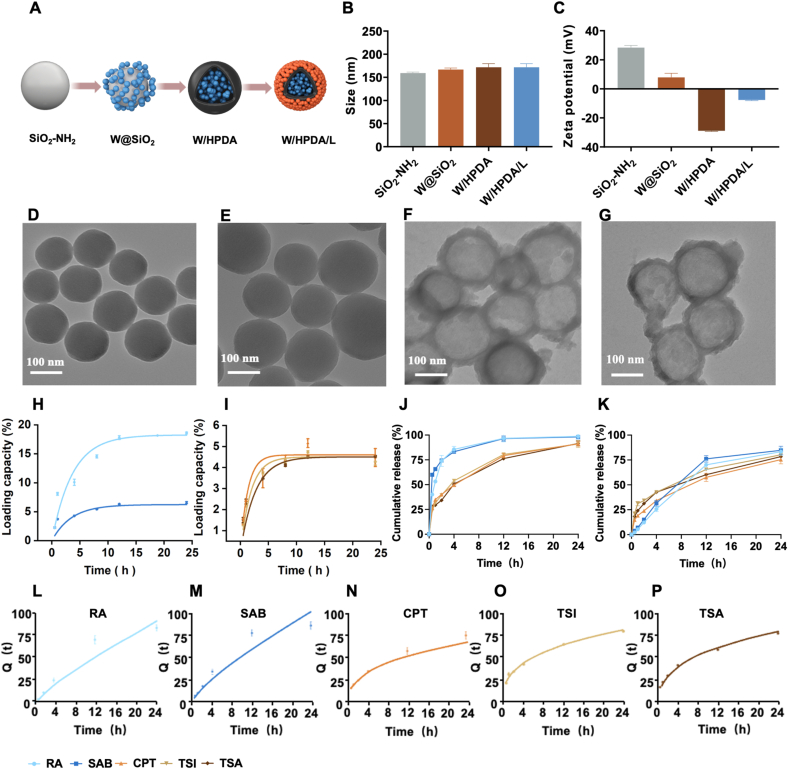
The preparation and characterization of W/HPDA/L. (A) Schematic illustration of W/HPDA/L NPs synthesis route. (B) Particle size and (C) Zeta potential of SiO_2_-NH_2_, W@SiO_2_, W/HPDA and W/HPDA/L. TEM image of SiO_2_-NH_2_ (D), W@SiO_2_ (E), W/HPDA (F) and W/HPDA/L (G). Adsorption kinetic model fitting for the adsorption of W (H) and L (I) on the adsorbent (pseudo-first-order). *In vitro* release profiles of five compounds from HPDA/WL (J) and W/HPDA/L (K) in 1 % SDS (pH 6.5–6.8) (n = 3). *In vitro* release model (Ritger-Peppas) curves of RA (L), SAB (M), CPT (N), TSI (O), and TSA (P) from W/HPDA/L. Data are presented as mean ± SD.

The synthesis protocol for W/HPDA/L nanoparticles is clearly illustrated in Fig. 2A. The process begins with the preparation of SiO_2_-NH_2_ nanoparticles using a straightforward self-polymerization method. This initial step involves the reaction of TEOS with APTES. W were co-incubated with SiO_2_-NH_2_ nanoparticles, which were then encapsulated with polydopamine. Subsequently, the SiO_2_-NH_2_ nanoparticles were etched, resulting in the distribution of W on the inner surface of HPDA. L were then co-incubated and adsorbed onto the outer surface of HPDA. The proportions of the five bioactive molecules were determined based on a comprehensive analysis of the Chinese Pharmacopoeia (ChP) standards and relevant *Salvia miltiorrhiza* research. The ChP stipulates that raw *Salvia miltiorrhiza* must contain ≥3.0 % SAB and ≥0.25 % combined CPT, TSI, and TSA, while its aqueous extract requires ≥0.5 % RA and ≥5.0 % SAB, and its alcohol extract requires ≥2.1 % CPT and ≥9.8 % TSA. Additionally, prior studies report that salvianolate for injection contains 2.68 % RA and 63.81 % SAB, and Danshen injection requires ≥0.1 mg/mL RA and ≥0.1 mg/mL SAB. Based on these standards and the actually measured contents, we selected an initial loading ratio of RA:SAB: CPT: TSI: TSA = 2:24:2:5:5 for W/HPDA/L@RBC-BOR preparation, which complies with ChP requirements [13,49,50]. The sequential, stratified loading of "cargos" is confirmed by the zeta potential (Fig. 2B) and dynamic light scattering data (Fig. 2C). As multiple drugs were loaded, the particle size of SiO_2_-NH_2_ nanoparticles increased from 159.23 ± 2.05 nm to 183.17 ± 4.67 nm. Changes in the nanoparticles' zeta potential correlate with the charges carried by both polydopamine (PDA) and the drugs. The loading of two distinct drug classes partially neutralized the charge carried by PDA nanoparticles. TEM images further revealed HPDA's hollow structure (Fig. 2D–G). For dual-component loading, we investigated the effects of drug-to-carrier mass ratio, reaction time, and temperature on DLE. For water-soluble components, increasing the mass ratio and prolonging the loading time enhanced drug loading until reaching plateaus at 1:1 ratio and 12 h, respectively. Elevated temperatures reduced loading efficiency, with 25 °C being optimal (Fig. S1A–C). Similar trends were observed for lipid-soluble components, which reached optimal loading at 0.5:1 ratio and 12 h, with 25 °C being the preferred temperature (Fig. S1D–F). After parameter optimization, the final DLE values for W and L in W/HPDA/L were 25.3 ± 1.21 % and 13.1 ± 2.03 %, respectively, demonstrating the excellent drug loading capacity of PDA nanoparticles.

This study investigated the adsorption mechanism of two classes of drugs within the PDA core. The adsorption kinetics of the drugs were fitted using pseudo-first-order and pseudo-second-order models. The resulting kinetic parameters are presented in Table 1. Fig. 2H and I display the pseudo-first-order kinetic model curves for RA, SAB, CPT, TSI, and TSA. The study results indicate that the loading kinetics of both drug categories align with the first-order kinetic model, suggesting that the drug loading process may be predominantly governed by physical adsorption [44].

**Table 1:** Kinetic fitting parameters.

Compound	Pseudo-first order			Pseudo-second order		
	Qe/(mg/g)	k	R^2^	Qe/(mg/g)	k	R^2^
RA	6.23	0.29	0.9706	7.78	0.03	0.8538
SAB	18.27	0.27	0.9764	21.95	0.01	0.8788
CPT	4.60	0.76	0.9768	5.06	0.14	0.8745
TSI	4.50	0.53	0.9896	5.21	0.16	0.8828
TSA	4.14	0.89	0.9766	4.74	0.21	0.8727

### 3.3 Release profiles of dual drug components from W/HPDA/L nanoparticles

Despite the distinct release rates of W and L in aqueous media due to their differing physicochemical properties, with W exhibiting a higher release rate than L, we strategically loaded these two components onto different regions of PDA nanoparticles (outer and inner) to achieve synchronous release. We then determined the release profiles of W and L. As illustrated in Fig. 2J–W and L in HPDA/WL group demonstrate markedly different release behaviors. RA and SAB released approximately 85.54 ± 3.07 % and 83.21 ± 0.18 % within the first 4 h, respectively, achieving almost complete release within 12 h. In contrast, only 25.71 ± 2.62 % of RA and 31.51 ± 2.87 % of SAB were released from W/HPDA/L nanoparticles within the first 4 h (Fig. 2K), indicating that encapsulation within PDA nanoparticles effectively delays the release of W. Conversely, the release profiles of lipid-soluble drugs remained minimal changes. This suggests that W and L positioned at different locations on PDA nanoparticles tend to be released synchronously and continuously.

The drug release mechanism was elucidated through fitting analysis of the Peppas equation. According to the Riger-Peppas equation for spherical core particles, the release mechanism is determined by the value of n: when n is less than 0.45, it indicates Fickian diffusion mode; when n is between 0.45 and 0.89, it suggests an anomalous transport mode, involving a combination of drug diffusion and polymer matrix erosion; when n is greater than 0.89, it indicates a polymer matrix erosion mode [51]. Fig. 2L–P illustrates the release fitting curves for the five components in W/HPDA/L, while Table 2 presents the fitted n values. The n values for L are all less than 0.45, indicating that the drug release pattern is dominated by Fick diffusion. In contrast, the n values for W fall between 0.45 and 0.89, suggesting that drug release may be influenced by both diffusion and polymer matrix dissolution. Based on these findings, it can be inferred that W gradually diffuse and release as the polydopamine core degrades, while L follow a Fickian diffusion mode of release on the carrier surface. Fickian diffusion, as a fundamental drug release mechanism driven by concentration gradients, remains unaffected by RBC membrane coating for lipophilic drugs in our study. The release profiles consistently followed Fick's laws, confirming that the membrane coating preserves the natural diffusion properties of these compounds.

**Table 2:** Release model parameters.

Model	RA	SAB	CPT	TSI	TSA
Ritger-Peppas	y = 6.28t^n^	y = 8.41t^n^	y = 19.26t^n^	y = 27.02t^n^	y = 24.68t^n^
	R^2^ = 0.9548	R^2^ = 0.9506	R^2^ = 0.9832	R^2^ = 0.9984	R^2^ = 0.9939
	n = 0.83	n = 0.78	n = 0.39	n = 0.35	n = 0.37

### 3.4 Preparation and characterization of W/HPDA/L@RBC-BOR

**Fig. 3:**
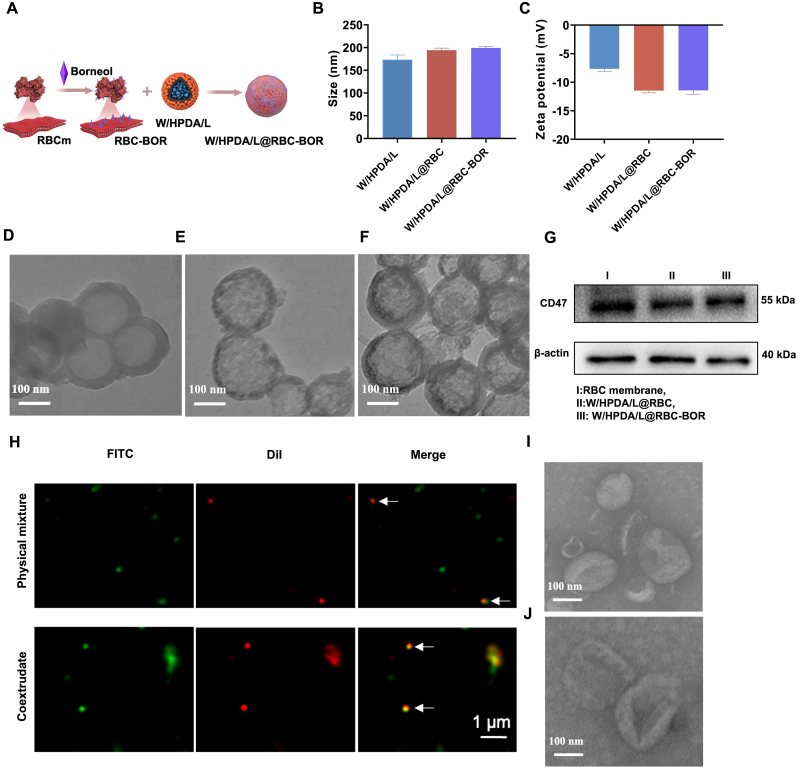
The preparation and characterization of W/HPDA/L@RBC-BOR. (A) Schematic illustration of W/HPDA/L@RBC-BOR synthesis route. (B) Particle size and (C) Zeta potential of W/HPDA/L, W/HPDA/L@RBC and W/HPDA/L@RBC-BOR (n = 3). TEM image of W/HPDA/L (D), W/HPDA/L@RBC (E) and W/HPDA/L@RBC-BOR (F). (G) Western blotting analysis of CD47 in RBC membrane, W/HPDA/L@RBC and W/HPDA/L@RBC-BOR. (H) Fluorescence colocalization of physical mixture and W/HPDA/L@RBC-BOR prepared by DiI-labeled RBC-BOR (red) and FITC-W/HPDA/L (green) was observed by LSCM. Scale bar: 1 μm. TEM image of RBC (I) and RBC-BOR (J). (For interpretation of the references to color in this figure legend, the reader is referred to the Web version of this article.)

To enhance the distribution of W/HPDA/L in the brain, we co-extruded the nanoparticles with red blood cell (RBC) membrane modified by borneol to fabricate W/HPDA/L@RBC-BOR (Fig. 3A). The resulting W/HPDA/L@RBC-BOR nanoparticles exhibited a diameter of 199.07 ± 3.09 nm and a zeta potential of −11.43 ± 0.67 mV. Compared to W/HPDA/L, both W/HPDA/L@RBC and W/HPDA/L@RBC-BOR showed an increase in particle diameter and a decrease in zeta potential (Fig. 3B and C), primarily due to the influence of the negatively charged cell membrane coating. Moreover, TEM results confirmed successful coating, as evidenced by a distinct shell visible on the surface of the W/HPDA/L core (Fig. 3D–F). While TEM images qualitatively demonstrated successful membrane coating on most nanoparticle surfaces, we quantitatively evaluated the coating efficiency using a fluorescence-based method. We labeled the PDA core with fluorescence and measured the quenching effect induced by membrane coating. As shown in Fig. S2A, the fluorescence intensity decreased progressively with increasing membrane input and eventually reached a plateau. Based on the fluorescence quenching data, we calculated that approximately 70 % of the W/HPDA/L core surface was effectively wrapped by RBC membranes through the extrusion process (Fig. S2B). The changes in particle size and zeta potential mutually confirm the fluorescence results (Fig. S2C–D).

Western blotting was used to determine the retention of CD47 in W/HPDA/L@RBC and W/HPDA/L@RBC-BOR (Fig. 3G). CD47 is a transmembrane protein expressed in RBC membranes that inhibits macrophage phagocytosis [52]. The results confirmed the presence of CD47 in both W/HPDA/L@RBC and W/HPDA/L@RBC-BOR. Fluorescence imaging co-localization can be employed to investigate the relative positions of the cell membrane and the core of nanoparticles (Fig. 3H). We labeled W/HPDA/L with FITC and RBC-BOR with DiI. In W/HPDA/L@RBC-BOR nanoparticles, the fluorescent signals of FITC and DiI showed significant overlap. In contrast, the physical mixture exhibited minimal yellow fluorescence. These findings collectively indicate that the cell membrane successfully envelops the surface of the carrier. Notably, the particle diameter and zeta potential of W/HPDA/L@RBC-BOR showed minimal changes compared to W/HPDA/L@RBC, which was further corroborated by TEM images before and after borneol modification (Fig. 3I and J). As shown in Fig. S3, the nanoparticle size distribution remained unchanged after 7 days of storage at 4 °C, with no significant aggregation or precipitation observed. The results demonstrate excellent storage stability of the W/HPDA/L@RBC-BOR nanoparticles. Furthermore, Fig. S4 confirms that the release profiles of both hydrophilic and hydrophobic components were well maintained after storage, showing comparable behavior to freshly prepared W/HPDA/L nanoparticles. These results collectively indicate that our nanoparticle formulation possesses satisfactory physical and chemical stability under refrigerated storage conditions. For the lipophilic components, their ability to freely permeate through the RBC membrane via passive diffusion was confirmed by their characteristic Fickian release profile, indicating minimal influence of the membrane coating on their release behavior. Regarding the hydrophilic components, although they cannot freely traverse the intact membrane, several factors contribute to maintaining their release efficiency: (1) the incomplete membrane coverage (∼70 % coating efficiency) leaves accessible channels; (2) protonation-induced membrane fusion/rupture under weakly acidic conditions further creates release pathways. Nevertheless, we did observe a moderate reduction in cumulative release for hydrophilic components after membrane coating (RA decreased from 83 % to 69 %; SAB from 84 % to 72 %), which we attribute to the partial masking effect of residual membrane structures.

### 3.5 Borneol reversibly enhances the *in vitro* BBB penetration of W/HPDA/L@RBC-BOR

**Fig. 4:**
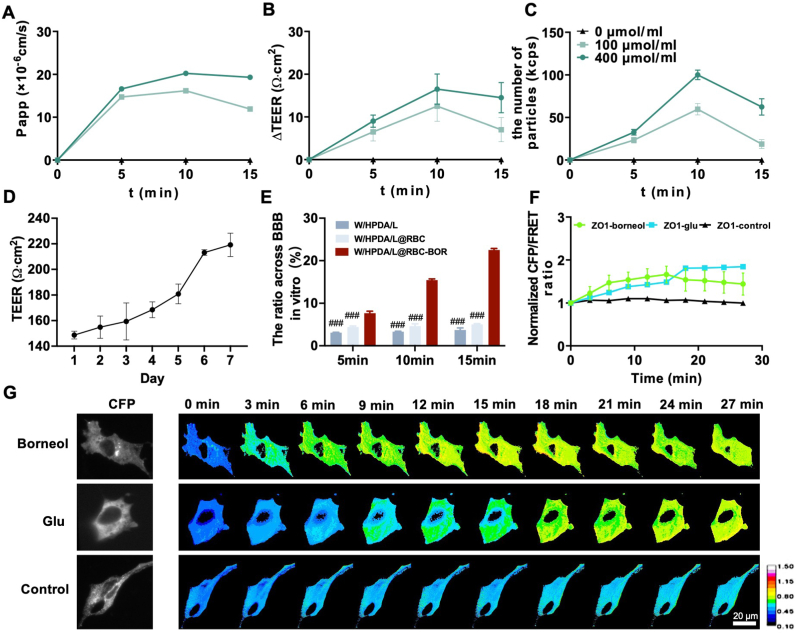
*In vitro* evaluation of the effect of borneol on the BBB. The impact of borneol on BBB permeability in changes in electrical resistance (A), permeability coefficients (B), and the number of particles passing through (C) (n = 3). (D) Transendothelial electrical resistance changes with time were monitored (n = 3). (E) The penetration efficiency of nanoparticles across the BBB at different times (n = 3). (F) Normalization of CFP/FRET signals corresponding to ZO1-tension vs time under borneol, Glu and Hanks stimulation (n = 3). (G) probe was treated with borneol, Glu and Hanks for 15 min and removed for another 15 min fluorescence images were processed using the 16-color map of Image J. Scale bar: 20 μm ^###^*P* < 0.001 *vs.* W/HPDA/L@RBC-BOR group. (For interpretation of the references to color in this figure legend, the reader is referred to the Web version of this article.)

Borneol is widely utilized in traditional Chinese medicine to enhance the distribution of central nervous system drugs in the brain due to its ability to open the BBB. However, the underlying mechanism remains unclear. To evaluate the effect of borneol on BBB permeability changes, including the dose-time-effect relationship, we employed a transwell model composed of bEnd.3 cells. Borneol demonstrated no cytotoxic effects on cells at concentrations ranging from 5 to 500 μmol/l (Fig. S5). We treated the bEnd.3 cell model with 0, 100 and 400 μmol borneol and assessed its impact on BBB permeability using three metrics: changes in electrical resistance, permeability coefficients, and the number of particles passing through. The results revealed that borneol's effect on improving BBB permeability exhibited both concentration and time dependence (Fig. 4A–C), with maximum BBB permeability reached at 10 min. During model construction, we continuously monitored the transepithelial electrical resistance (TEER) of the cell layer. A resistance exceeding 200 Ω cm^2^ indicated successful establishment of the *in vitro* BBB model (Fig. 4D). We evaluated the improved ability of borneol-containing formulations to cross the BBB using an *in vitro* model. As shown in Fig. 4E, the permeability of W/HPDA/L@RBC-BOR was 6.07-fold and 4.48-fold higher than that of W/HPDA/L and W/HPDA/L@RBC, respectively. These findings indicate a significant enhancement in the penetration efficiency of nanoparticles across the BBB upon the addition of borneol.

Stabilization of the BBB structure is closely related to the tight binding of protein ZO1 [53]. We constructed FRET-based tension probes of the transmembrane protein ZO1 to visualize the changes of protein ZO1 [46]. Therefore, changes of ZO1 tension indicate changes of permeability in the BBB. Glutamate (Glu), commonly used to establish BBB injury models, destabilizes BBB structure and increases its permeability [54]. Fig. 4F and G, upon addition of Glu to the cells, ZO1 tension increased and continued to rise even 15 min after Glu removal. Conversely, when borneol was added to the cells, ZO1 tension increased but decreased after 15 min of borneol removal. Treatment with Hanks' solution showed minimal change in ZO1 tension. These data suggest that Glu-induced BBB opening elevates ZO1 tension, and upon drug withdrawal, further elevation in ZO1 tension may indicate sustained open injury. In contrast, borneol's effect on opening the BBB is reversible. Therefore, the borneol in W/HPDA/L@RBC-BOR demonstrates a certain level of safety in terms of its ability to increase BBB permeability, as its effects are reversible.

### 3.6 W/HPDA/L@RBC-BOR significantly suppresses ROS and inflammation levels *in vitro*, exerting neuroprotective effects

**Fig. 5:**
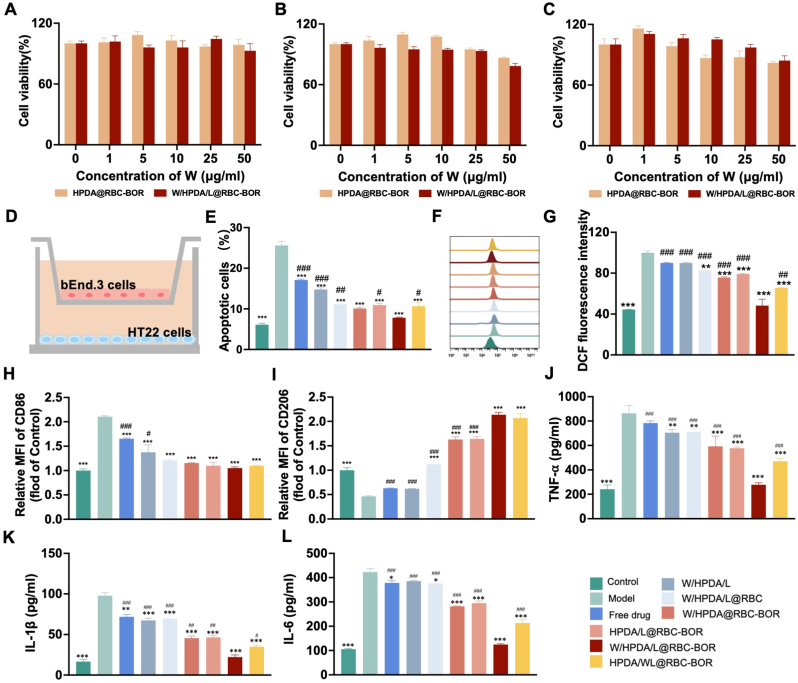
The protective effect of W/HPDA/L@RBC-BOR *in vitro*. Cell viability of BV2 cells (A), HT22 cells (B) and bEnd.3 cells (C) incubated with different doses of HPDA@RBC-BOR and W/HPDA/L@RBC-BOR (n = 5). (D) Schematic diagram of cell model *in vitro*. (E) Semiquantitative results of cell apoptosis in each group (n = 3). Flow cytometry images (F) and quantitative analysis (G) of DCFH-DA staining to monitor intracellular ROS level (n = 3). (H) Semiquantitative results of CD86 MFI in each group (n = 3). (I) Semiquantitative results of CD206 MFI in each group (n = 3). Determination of inflammatory factors as TNF-α (J), IL-1β (K), and IL-6 (L) in BV2 cells (n = 3). Data are presented as mean ± SD. ∗*P* < 0.05, ∗∗*P* < 0.01, ∗∗∗*P* < 0.001 *vs*. model; ^#^*P* < 0.05, ^##^*P* < 0.01, ^###^*P* < 0.001 *vs*. W/HPDA/L@RBC-BOR group.

The cytotoxicity of W/HPDA/L@RBC-BOR was initially evaluated in HT22, BV2, and bEnd.3 cells. As illustrated in Fig. 5A–C, both HPDA@RBC-BOR and W/HPDA/L@RBC-BOR exhibited no discernible cytotoxic effects across a concentration range of 1–50 μg/mL (based on water-soluble ingredient concentration) on HT22, BV2, and bEnd.3 cells, indicating the safety of these particles for subsequent *in vitro* studies. To assess the potential neuroprotective effects of W/HPDA/L@RBC-BOR, a transwell model combined with an OGD/R model mimicking ischemic stroke conditions was employed *in vitro*, as shown in Fig. 5D. Following OGD/R exposure, the apoptosis rate of HT22 cells in the model group increased approximately 4.18-fold (25.61 %) compared to the physiological saline group. Representative images of cell apoptosis analyzed with flow cytometry can be seen in Fig. S6. Treatment with W/HPDA@RBC-BOR, HPDA/L@RBC-BOR, and HPDA/WL@RBC-BOR resulted in HT22 cell apoptosis rates of 10.08 ± 0.35 %, 10.91 ± 0.74 %, and 10.67 ± 1.67 %, respectively. Notably, treatment with W/HPDA/L@RBC-BOR nanospheres further reduced the apoptosis rate of HT22 cells to 7.81 ± 0.24 %, lower than that observed with W/HPDA@RBC-BOR, HPDA/L@RBC-BOR, or HPDA/WL@RBC-BOR (Fig. 5E). These results suggest that both W and L can exert neuroprotective effects to some extent. Their combined use demonstrates a more potent pharmacological effect, potentially indicating a synergistic relationship.

Oxidative stress and elevated levels of reactive oxygen species (ROS) during ischemic injury significantly impair neuronal survival. Prompt ROS clearance is a crucial strategy for mitigating acute ischemia-reperfusion injury. In this study, we utilized flow cytometry to evaluate the antioxidant therapeutic effects of various formulations by measuring ROS levels in HT22 cells. Compared to the physiological saline group, the model group exhibited a marked increase in ROS fluorescence intensity, confirming successful modeling. ROS levels in all treatment groups (W/HPDA/L@RBC, W/HPDA@RBC-BOR, HPDA/L@RBC-BOR, W/HPDA/L@RBC-BOR, and HPDA/WL@RBC-BOR) were significantly lower than in the model group, with reductions of 17.45 ± 0.61 %, 24.25 ± 2.45 %, 20.84 ± 2.29 %, 51.74 ± 10.96 %, and 34.43 ± 3.63 %, respectively (Fig. 5F and G). Notably, the W/HPDA/L@RBC-BOR group demonstrated the highest ROS scavenging capacity. This finding underscores the superiority of multi-component combinations over single-component therapy. Moreover, the simultaneous release of various drugs further enhanced antioxidant capacity, suggesting a synergistic effect in combating oxidative stress.

In addition to oxidative stress, inflammation plays a pivotal role in ischemia-reperfusion. Damaged neurons release danger-associated molecular patterns, activating and recruiting microglial cells. Activated microglia are divided into M1 (pro-inflammatory) and M2 (anti-inflammatory) types. M1 microglia produce pro-inflammatory factors such as TNF-α, IL-1β, and IL-6, exacerbating neuroinflammation and damage. In contrast, M2 microglia secrete anti-inflammatory factors like IL-10 and TGF-β, promoting neuronal repair and regeneration. Therefore, promoting the polarization of microglia from M1 to M2 phenotype can attenuate the inflammatory response and salvage injured neurons [[55], [56], [57]]. We treated OGD/R-exposed microglia with different formulations and monitored their status by examining the expression of M1 marker CD86 and M2 marker CD206. Results showed that OGD/R significantly increased CD86 expression and decreased CD206 expression in the model group. Treatment with various formulations reversed this trend, decreasing CD86 expression while increasing CD206 expression (Fig. 5H and I). Specifically, CD86 expression decreased by 42.43 ± 0.47 %, 45.17 ± 0.42 %, 47.74 ± 4.86 %, 50.05 ± 1.64 %, and 47.89 ± 0.28 % in the W/HPDA/L@RBC, W/HPDA@RBC-BOR, HPDA/L@RBC-BOR, W/HPDA/L@RBC-BOR, and HPDA/WL@RBC-BOR groups, respectively. Concurrently, CD206 expression increased 2.41, 3.51, 3.53, 4.61, and 4.45-fold compared to the model group. These results indicate that both W and L can significantly promote M1 to M2 microglial polarization, with a more pronounced effect observed in their combined administration. The phenotypic transition of microglia influences pro-inflammatory cytokine expression. Analysis of inflammatory cytokines TNF-α, IL-1β, and IL-6 revealed that all formulations significantly reduced their expression compared to the model group, indicating inflammation alleviation. Notably, the W/HPDA/L@RBC-BOR group demonstrated the most substantial reductions of 67.75 ± 3.31 %, 77.28 ± 5.11 %, and 70.48 ± 0.22 % in TNF-α, IL-1β, and IL-6 expression, respectively, significantly outperforming other groups (Fig. 5J–L). These findings suggest that the combined administration of W and L effectively mitigates inflammation during ischemia-reperfusion, potentially through synergistic effects on microglial polarization and cytokine suppression.

### 3.7 W/HPDA/L@RBC-BOR exhibited enhanced bioavailability and synchronized drug release in SD rats

**Fig. 6:**
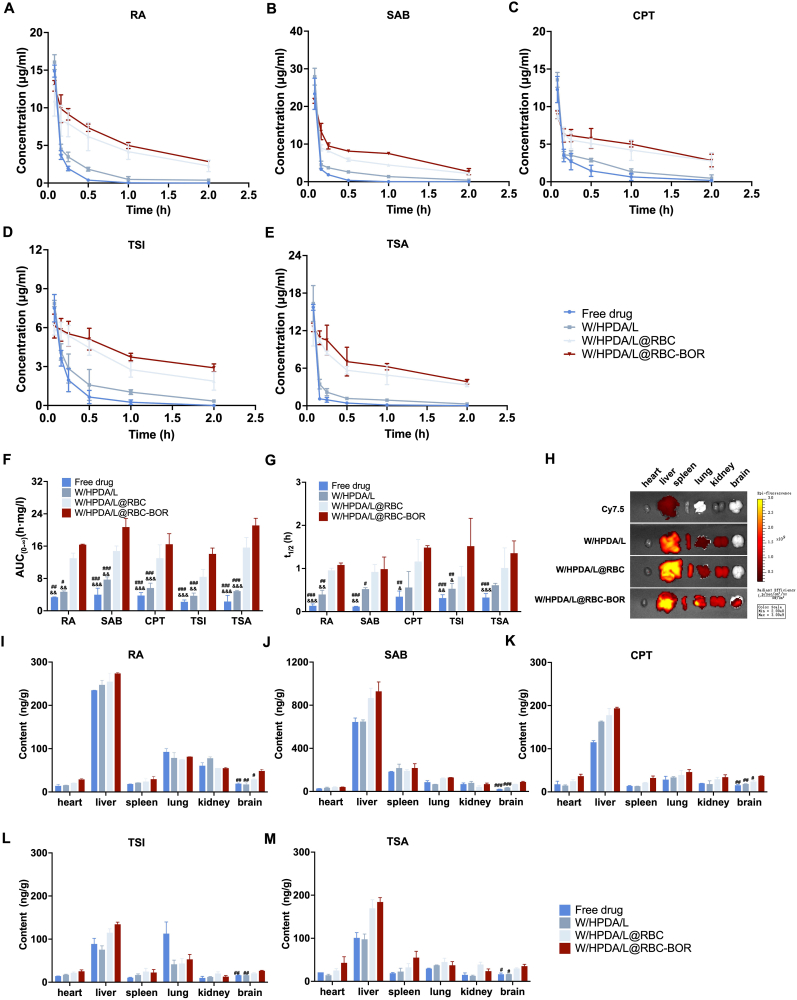
Pharmacokinetics and tissue distribution of W/HPDA/L@RBC-BOR. Mean drug concentration–time curve of RA(A), SAB(B), CPT(C), TSI(D) and TSA(E) in the plasma of rats after free drug, W/HPDA/L, W/HPDA/L@RBC and W/HPDA/L@RBC-BOR injection. AUC_(0-∞) (_F) and t_1/2_(G) values of the five compounds. (H) Representative *ex vivo* fluorescence imaging of the brain, heart, lung, liver, kidney, and spleen of mice at 8 h after administration of cy7.5, W/HPDA/L, W/HPDA/L@RBC and W/HPDA/L@RBC-BOR. Quantification of RA(I), SAB(J), CPT(K), TSI(L) and TSA(M) accumulation in major organs excised from mice 12 h post tail vein injection of with free drug, W/HPDA/L, W/HPDA/L@RBC and W/HPDA/L@RBC-BOR. Data are presented as mean ± SD (n = 3). ^&^*P* < 0.05, ^&&^*P* < 0.01, ^&&&^*P* < 0.001 *vs*. W/HPDA/L@RBC group; ^#^*P* < 0.05, ^##^*P* < 0.01, ^###^*P* < 0.001 *vs*. W/HPDA/L@RBC-BOR group.

Employing SD rats as our animal model, we evaluated the *in vivo* pharmacokinetic profiles of various Danshen nanoparticle formulations. As illustrated in Fig. 6A–F and detailed in Tables S2–6, both RBC membrane-modified nanoparticles, W/HPDA/L@RBC and W/HPDA/L@RBC-BOR, exhibited extended circulation times and enhanced bioavailability compared to their free drug counterparts. The modification of the cell membrane surface with borneol did not significantly affect the *in vivo* distribution behavior of the drugs. This unexpected result could be explained by the fact that the brain, despite being the target organ, represents only a small fraction of the total drug distribution throughout the body. Consequently, any changes in brain uptake facilitated by borneol might not be prominent enough to significantly impact the overall pharmacokinetic parameters observed in systemic circulation. Notably, within the W/HPDA/L@RBC-BOR group, the half-lives (t_1/2_) of the water-soluble constituents, RA and SAB, were substantially prolonged by 8.01-fold and 8.49-fold, respectively. These increases significantly outpaced those observed in the lipid-soluble components: TSI (4.29-fold), TSA (4.83-fold), and CPT (4.16-fold). This disparity suggests a decelerated drug release behavior for compounds encapsulated within the inner PDA layer. Comparative analysis with the free drug group revealed that the half-lives of the two distinct drug types (W and L) converged more closely within the W/HPDA/L@RBC-BOR group (Fig. 6G). This convergence indicates a synchronous release of both water-soluble and lipid-soluble constituents by the novel formulation, aligning with the *in vitro* release profiles previously observed.

### 3.8 W/HPDA/L@RBC-BOR demonstrates excellent brain-targeting properties in SD rats

To evaluate the brain-targeting properties of W/HPDA/L@RBC-BOR, we intravenously administered W/HPDA/L, W/HPDA/L@RBC, or W/HPDA/L@RBC-BOR (containing equimolar cy7.5-labeled W/HPDA/L) to SD rats, followed by *ex vivo* fluorescence imaging. *Ex vivo* imaging of brains after 4, 8 and 12 h treatment of W/HPDA/L@RBC-BOR exhibited the strongest fluorescence accumulation (Fig. S7). Of the three time points, the strongest fluorescence signal in all groups seemed to come from 8 h after injection (Fig. 6H). Further quantification of drug distribution in tissues using UPLC-MS/MS revealed significantly higher levels of drugs in the brain for W/HPDA/L@RBC-BOR compared to free drugs and W/HPDA/L (Fig. 6I–M). This enhanced brain accumulation can be attributed to two factors: 1) the increased BBB permeability induced by BOR; 2) the biomimetic effect of red blood cell membrane modification, which prolongs the circulation time of the formulation *in vivo*. These factors synergistically contribute to the enhanced drug accumulation in the brain. While extended half-lives should theoretically enhance sustained target exposure, we recognize that multiple factors may contribute to the observed 1.6-fold difference in brain accumulation, including differential BBB transport efficiency and possible efflux mechanisms that may affect the components differently. Therefore, the drug half-life cannot be directly correlated with the degree of drug accumulation in the brain at a specific time point.

### 3.9 W/HPDA/L@RBC-BOR demonstrates significant therapeutic efficacy against ischemic stroke in in vivo experiments

**Fig. 7:**
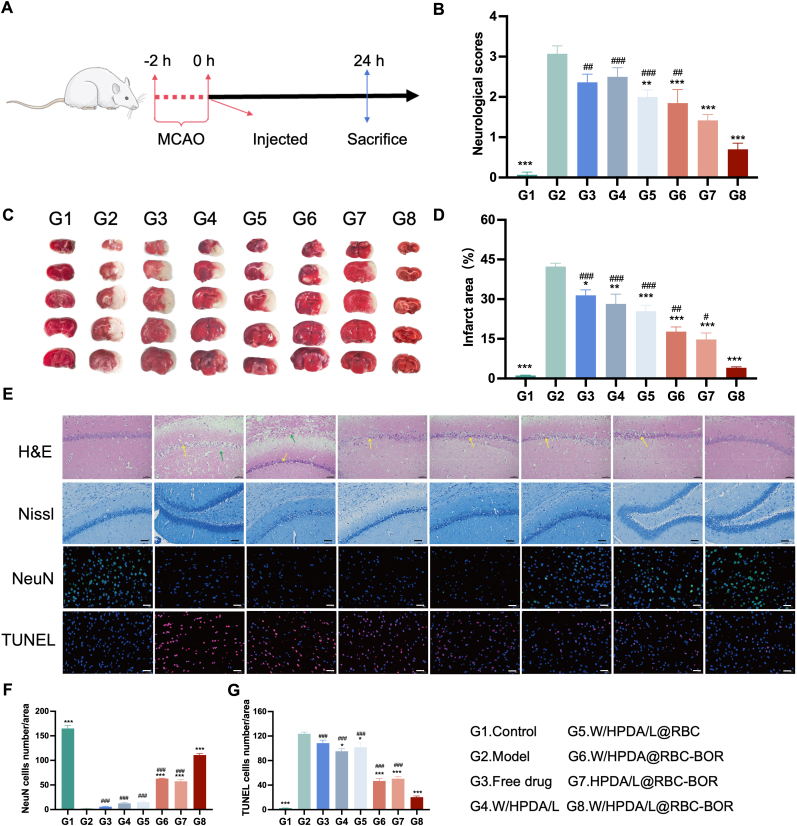
*In vivo* anti-ischemic stroke efficacy. (A) Scheme of the animal experiment in the MCAO/R model. (B) neurological scores of different treatment groups. (C) Images of TTC staining with different treatments. (D) Quantitative analysis of the ratio of the infarct volume to the total brain volume. (E) Histological evaluation of brain sections by H&E, Nissl, TUNEL and NeuN staining when rats after MCAO/R treatments were administered with different nanospheres. Scale bar: 50 μm. (F–G) Assessment of neuronal apoptosis by NeuN and TUNEL staining. Data are presented as mean ± SD (n = 6). ∗*P* < 0.05, ∗∗*P* < 0.01, ∗∗∗*P* < 0.001 *vs*. model; ^#^*P* < 0.05, ^##^*P* < 0.01, ^###^*P* < 0.001 *vs*. W/HPDA/L@RBC-BOR group.

The anti-ischemic stroke effects of various drug formulations were evaluated using an established MCAO/R model, with the protocol illustrated in Fig. 7A. Neuro scores and infarct areas were assessed 24 h post-ischemic reperfusion (Fig. 7B–D). TTC staining revealed that the infarct area reached 42.33 ± 3.11 % of the total brain tissue after MCAO/R. Significant reductions in brain tissue damage were observed in the W/HPDA/L@RBC, W/HPDA@RBC-BOR, HPDA/L@RBC-BOR, and W/HPDA/L@RBC-BOR groups, with infarct areas of 25.46 ± 5.46 %, 17.74 ± 4.31 %, 14.74 ± 6.03 %, and 4.06 ± 0.77 %, respectively. The W/HPDA/L@RBC-BOR treatment group exhibited the smallest infarct area, indicating its superior protective efficacy. Further investigation of neuro scores to evaluate rats' neurological functions yielded findings largely consistent with the infarct area results. Compared to the model group, all formulations containing various constituents ameliorated ischemia-reperfusion-induced neurological deficits to varying degrees. Notably, the borneol-modified formulation (W/HPDA/L@RBC-BOR) demonstrated significantly greater efficacy than its unmodified counterpart (W/HPDA/L@RBC), attributable to borneol's enhancement of brain drug delivery efficiency. Moreover, formulations containing both lipophilic and hydrophilic components exhibited superior efficacy compared to those with only a single class of component (W/HPDA@RBC-BOR and HPDA/L@RBC-BOR). This observation suggests a synergistic effect between the two classes of drugs in the stroke process, highlighting the advantage of synchronized release.

To further validate our findings, we employed four histological staining techniques—H&E, Nissl, TUNEL, and NeuN—to visualize cortical tissue and neuronal morphology. H&E and Nissl staining revealed disordered organization, edema, loosened cytoplasm, and neuronal necrosis in the peri-infarcted cortex of MCAO/R rats, along with a reduction in neuronal Nissl bodies (Fig. 7E). Following treatment with six different Danshen formulations, damage in the ischemic regions was reversed, with W/HPDA/L@RBC-BOR demonstrating particularly superior efficacy. NeuN/TUNEL staining was utilized to visually assess neuronal apoptosis post-MCAO/R in rats, with staining results quantified using ImageJ software. The MCAO/R group exhibited a significant increase in TUNEL-positive cells compared to the saline group. Notably, the W/HPDA@RBC-BOR, HPDA/L@RBC-BOR, and W/HPDA/L@RBC-BOR groups significantly restored neuronal signaling and reduced apoptosis. It is particularly noteworthy that treatment with W/HPDA/L@RBC-BOR nanoparticles demonstrated the most potent apoptosis inhibition effect. These findings align with the results from our anti-ischemic stroke efficacy experiments (Fig. 7F and G).

**Fig. 8:**
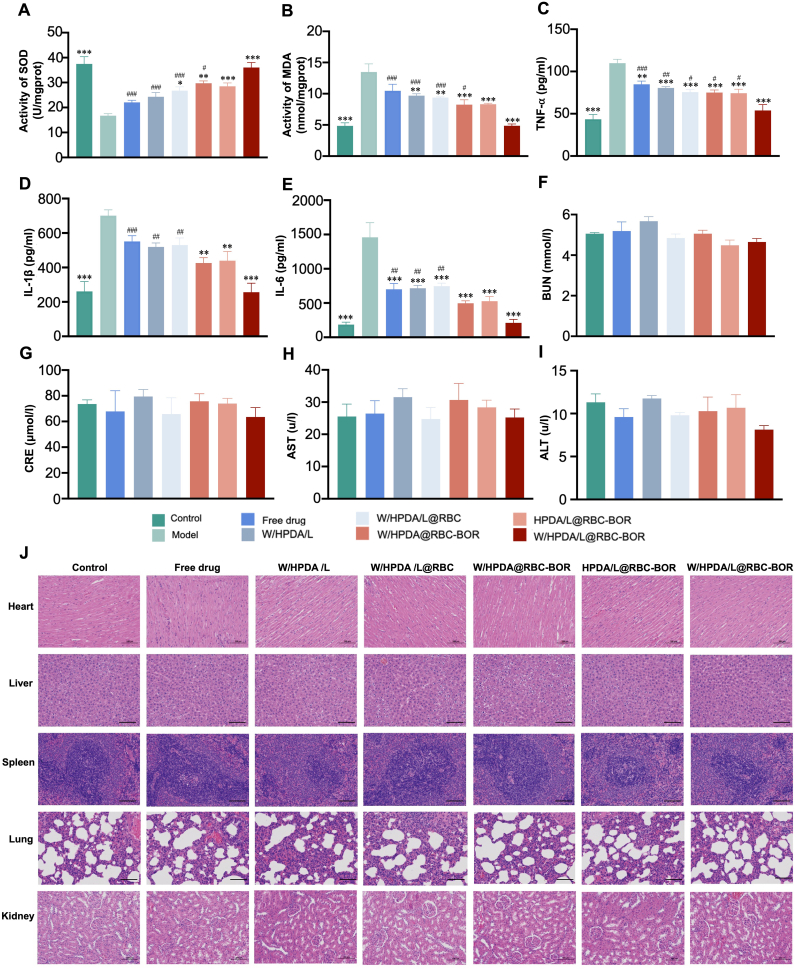
The expression of oxidative stress index as SOD (A) and MDA (B) in brain tissue after cerebral ischemic-reperfusion. The production of inflammatory factors as TNF-α (C), IL-1β (D), and IL-6 (E) in the ischemic hemisphere. Blood chemistry analysis of the mice including BUN (F), CRE (G), AST (H) and ALT (I). (J)H&E staining of main organs. Scale bars, 100 μm. Data are presented as mean ± SD (n = 5). ∗*P* < 0.05, ∗∗*P* < 0.01, ∗∗∗*P* < 0.001 *vs*. model; ^#^*P* < 0.05, ^##^*P* < 0.01, ^###^*P* < 0.001 *vs*. W/HPDA/L@RBC-BOR group.

We then investigated the antioxidant capacity of W/HPDA/L@RBC-BOR by examining indicators related to oxidative stress. Treatment with W/HPDA/L@RBC-BOR effectively alleviated oxidative stress in the ischemic hemisphere, reducing MDA levels by 63.94 ± 5.85 % and increasing SOD expression by 115.74 ± 8.43 % (Fig. 8A and B). Intracerebral inflammatory factor levels of TNF-α, IL-1β, and IL-6 were further quantified using ELISA kits. As shown in Fig. 8C–E, pro-inflammatory cytokine content in the brains of MCAO/R rats increased significantly. W/HPDA/L@RBC-BOR treatment produced the most potent anti-inflammatory effect, decreasing the expression of pro-inflammatory factors TNF-α, IL-1β, and IL-6 by 50.82 ± 6.69 %, 63.19 ± 8.87 %, and 85.62 ± 4.18 %, respectively. These data demonstrate that W/HPDA/L@RBC-BOR can effectively mitigate oxidative stress and inhibit inflammation in the ischemic microenvironment. The potential toxicity of W/HPDA/L@RBC-BOR during treatment was evaluated through serum biochemical analysis of hepatic and renal function indices, as well as H&E staining of major organs. Serum chemistry tests (BUN, CRE, AST, and ALT) for renal and hepatic function analysis showed no significant abnormalities (Fig. 8F–I). Furthermore, H&E staining results revealed no major abnormalities in tissue sections, indicating the absence of organ toxicity following intravenous administration of various formulations (Fig. 8J). We have conducted comprehensive toxicity studies over 7-day and 21-day periods. The histological analysis of major organs (heart, liver, spleen, lung, kidney and brain) revealed no pathological abnormalities, including no evidence of necrosis, inflammation, or fibrosis, confirming the excellent biocompatibility and long-term safety profile of W/HPDA/L@RBC-BOR for potential therapeutic applications. (Fig. S8).

### 3.10 W/HPDA/L@RBC-BOR exert neuroprotective effects in stroke through the TNFα and NF-κB pathways

**Fig. 9:**
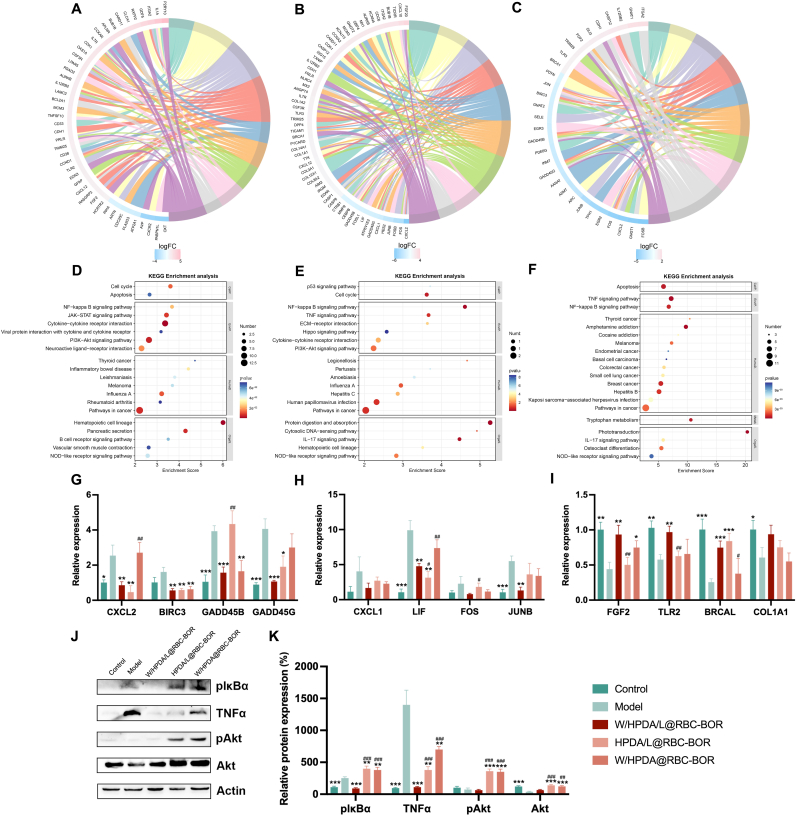
The mechanism of W/HPDA/L@RBC-BOR on ischemic stroke. GO analyses of differentially expressed genes regulated by (A) W/HPDA@RBC-BOR, (B) HPDA/L@RBC-BOR and (C) W/HPDA/L@RBC-BOR injection. KEGG analyses of differentially expressed genes regulated by (D) W/HPDA@RBC-BOR, (E) HPDA/L@RBC-BOR and (F) W/HPDA/L@RBC-BOR injection. RT-PCR analysis of differentially gene expression levels related to NF-κB signaling pathway (G), TNF signaling pathway (H) and PI3K-Akt signaling pathway (I). (J–K) The expression levels of plκBα, TNFα, pAkt and Akt protein were assessed by Western blotting. Data are presented as mean ± SD (n = 3). ∗*P* < 0.05, ∗∗*P* < 0.01, ∗∗∗*P* < 0.001 *vs.* model; ^*#*^*P* < 0.05;^*##*^*P* < 0.01, ^*###*^*P* < 0.001 vs. W/HPDA/L@RBC-BOR group.

To further elucidate the mechanism underlying the therapeutic effects of W/HPDA/L@RBC-BOR on ischemic stroke, we conducted RNA sequencing analysis to examine gene expression regulation and analyze differentially expressed genes. Comparative analysis revealed that, compared to the model group, the W/HPDA@RBC-BOR group upregulated 272 genes and downregulated 146 genes; the HPDA/L@RBC-BOR group upregulated 476 genes and downregulated 181 genes; and the W/HPDA/L@RBC-BOR group upregulated 129 genes and downregulated 135 genes (Fig. S9). To enhance relevance to ischemic stroke pathology, differentially expressed genes from each group were matched across multiple omics platforms using STROMICS, followed by GO and KEGG enrichment analysis for W/HPDA@RBC-BOR (Fig. 9A and D), HPDA/L@RBC-BOR (Fig. 9B and E), and W/HPDA/L@RBC-BOR groups (Fig. 9C and F).

The key genes identified in these analyses suggest that W/HPDA@RBC-BOR, HPDA/L@RBC-BOR, and W/HPDA/L@RBC-BOR act on various intracellular signaling pathways, potentially involving NF-κB, TNF, and PI3K-Akt pathways associated with cell apoptosis and survival, which may play a role in the treatment of ischemic stroke. NF-κB, a protein complex, is a crucial nuclear transcription factor involved in cellular responses to stimuli such as inflammation, immune responses, and regulation of cell apoptosis and stress responses. Excessive activation of NF-κB has been associated with inflammatory changes in various human diseases, including brain disorders. In neurodegenerative diseases, dysfunction of the PI3K/AKT signaling pathway leads to disturbances in cell survival and synaptic function. Modulation of the PI3K/AKT signaling pathway can restore neuronal function and alleviate disease symptoms, including mitigating oxidative stress-induced damage to neurons. Therefore, the co-delivery of two components from Danshen may exert neuroprotective effects through multiple pathways. To further elucidate these predicted mechanisms, we conducted quantitative polymerase chain reaction (qPCR) to measure the expression levels of 16 genes. As depicted in Fig. 9G–I, compared to the model group, the W/HPDA/L@RBC-BOR group significantly downregulated gene expression of CXCL2, BIRC3, GADD45B, and GADD45G in the NF-κB signaling pathway, while also downregulating gene expression of CXCL1, LIF, FOS, and JUNB in the TNF signaling pathway. Additionally, the W/HPDA/L@RBC-BOR group significantly upregulated gene expression of FGF2, TLR2, BRCAL, and COL1A1 in the PI3K/AKT pathway, consistent with the transcriptomic findings.

Based on differential transcriptomics results, we hypothesize that W and L of Danshen may exert synergistic effects through the PI3K-AKT pathway, TNF-α pathway, or NF-κB pathway. Therefore, we utilized Western Blot experiments to separately examine the impact of W/HPDA/L@RBC-BOR, W/HPDA@RBC-BOR, and HPDA/L@RBC-BOR on the expression levels or phosphorylation levels of key proteins within these pathways to determine their regulatory effects (Fig. 9J and K). IκB (Inhibitor of kappa B) is a critical component of the NF-κB signaling pathway. NF-κB is a group of transcription factors that, in a resting state, are bound by the inhibitory protein IκB and retained in the cytoplasm, preventing their entry into the nucleus. Phosphorylation of IκB leads to its ubiquitination and degradation, thereby activating the NF-κB pathway. Thus, detecting the phosphorylation level of IκB can reflect the activation status of the NF-κB pathway in cells. The results showed that, compared to healthy mice, the TNFα and NF-κB pathways were significantly activated in the tissues of the model group mice. W and L of Danshen did not significantly inhibit the NF-κB pathway, whereas W/HPDA/L@RBC-BOR significantly inhibited the NF-κB pathway, suggesting a synergistic effect between W and L. Additionally, both W and L of Danshen were able to inhibit the TNFα pathway, and their combined use showed an even more pronounced inhibitory effect on this pathway, indicating that the combination enhances the inhibition of the TNFα pathway. On the other hand, W and L of Danshen significantly enhanced the activation of the PI3K-AKT pathway in tissues, but no synergistic effect was observed when used together. Therefore, W and L of Danshen exert neuroprotective effects in stroke through the TNFα and NF-κB pathways.

## 4 Conclusion

In conclusion, our study presents an innovative brain-targeted biomimetic delivery system, co-loading five distinct components of Danshen for the synergistic treatment of ischemic stroke. Utilizing hollow polydopamine nanoparticles coated with red blood cell membranes and modified with borneol, we achieved synchronized release and enhanced BBB penetration. Our experimental results demonstrate that in oxygen-glucose deprivation/reoxygenation (OGD/R) models mimicking ischemic conditions, treatment with W/HPDA/L@RBC-BOR reduced apoptosis rates in HT22 cells to 7.81 ± 0.24 %. This indicates a potent neuroprotective effect due to the synergistic action of the co-loaded components. Further, flow cytometry analysis showed that W/HPDA/L@RBC-BOR had the highest ROS scavenging capacity, reducing ROS levels by 51.74 ± 10.96 %. Inflammation markers were also markedly reduced, with TNF-α, IL-1β, and IL-6 levels decreasing by 67.75 ± 3.31 %, 77.28 ± 5.11 %, and 70.48 ± 0.22 % respectively, highlighting the formulation's efficacy in mitigating inflammation. The brain-targeting efficiency was significantly improved, with a 1.6-fold increase in brain fluorescence intensity compared to non-modified formulations, demonstrating effective accumulation at the target site. *In vivo* animal experiments have also confirmed that the synchronized release of both W and L of Danshen demonstrates superior therapeutic effects for ischemic stroke compared to W alone, L alone, or a simple mixture of W and L. Subsequent mechanistic studies have shown that W and L exert their combined therapeutic effect by synergistically inhibiting the NF-κB and TNFα pathways in neurons, thereby improving cellular inflammation levels. Overall, this study not only provides a promising approach for the treatment of ischemic stroke but also paves the way for future research in multi-modal therapies for complex neurological disorders. The combination of advanced nanotechnology and traditional Chinese medicine components offers a novel and effective strategy to enhance therapeutic outcomes and improve patient prognosis.

## CRediT authorship contribution statement

**Chenjie Xia:** Writing – original draft, Visualization, Methodology, Investigation, Data curation, Conceptualization. **Changhui Hu:** Methodology, Investigation, Data curation. **Rui Xu:** Methodology. **Feihong Zhuo:** Methodology. **Mengfei Yang:** Methodology. **Yinjia Li:** Methodology. **Zixuan Shan:** Methodology. **Cheng Xu:** Methodology. **Yutong Wang:** Writing – review & editing, Supervision, Project administration. **Zhipeng Chen:** Writing – review & editing, Supervision, Resources, Project administration, Funding acquisition, Conceptualization.

## Declaration of competing interest

The authors declare that they have no known competing financial interests or personal relationships that could have appeared to influence the work reported in this paper.

## Data Availability

Data will be made available on request.
