# Supplementary material for: An efficient brain delivery system co-loaded with multiple components of Salvia miltiorrhiza for synergistic treatment of ischemic stroke
Source: Mater Today Bio. 2025 Jul 16;34:102102. doi: 10.1016/j.mtbio.2025.102102 (PMC12311606; doi:10.1016/j.mtbio.2025.102102)
Supplement: Multimedia component 1 [file mmc1.docx]

**Supplementary Material**

**An efficient brain delivery system co-loaded with multiple components of** ***Salvia miltiorrhiza* for synergistic treatment of ischemic stroke**


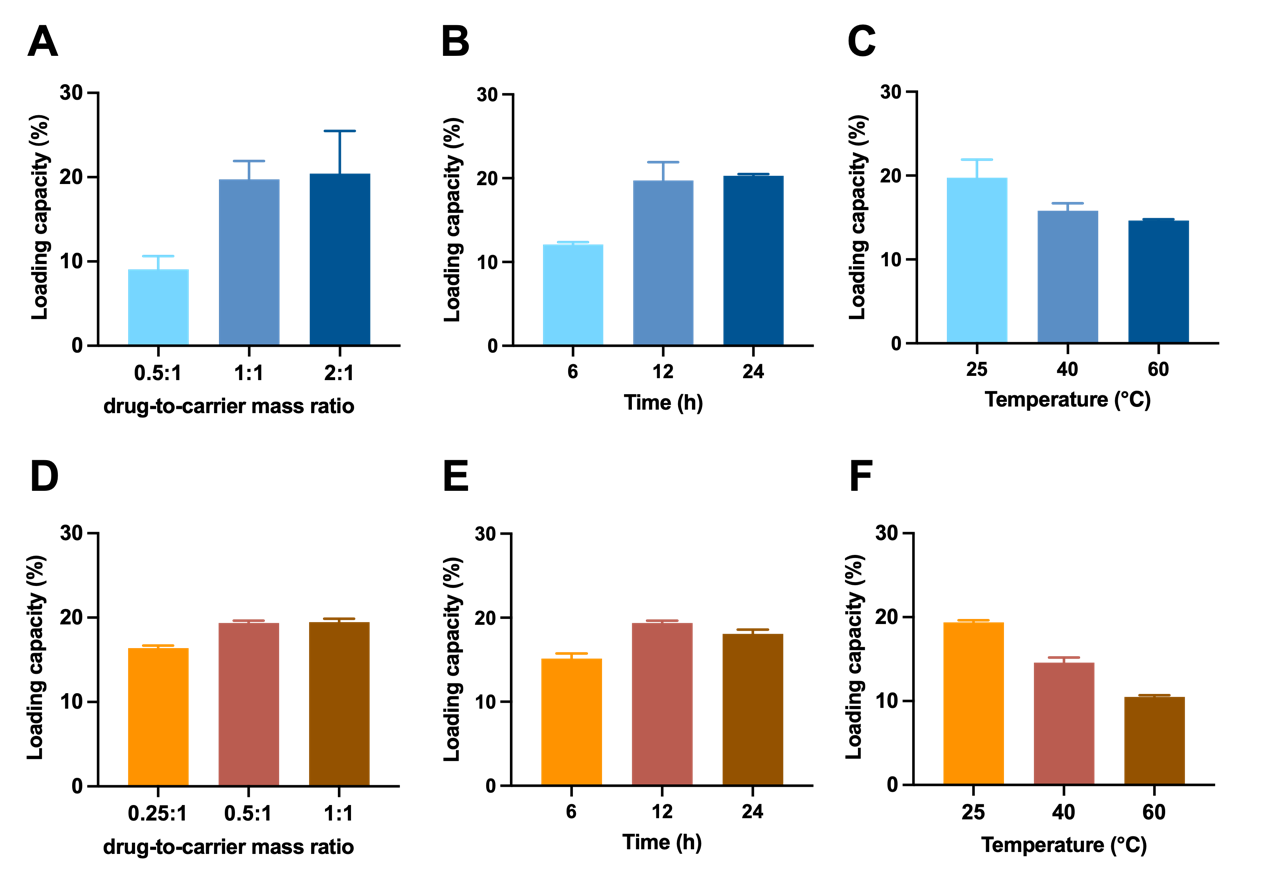


Figure S1. Process optimization of W/HPDA/L Nanoparticles. The effects of mass ratio of W to carrier (A), drug loading time (B), and temperature (C) on the drug loading capacity of W (n=3). The effects of mass ratio of L to carrier (D), drug loading time (E), and temperature (F) on the drug loading capacity of L. Data are presented as mean ± SD (n=3).


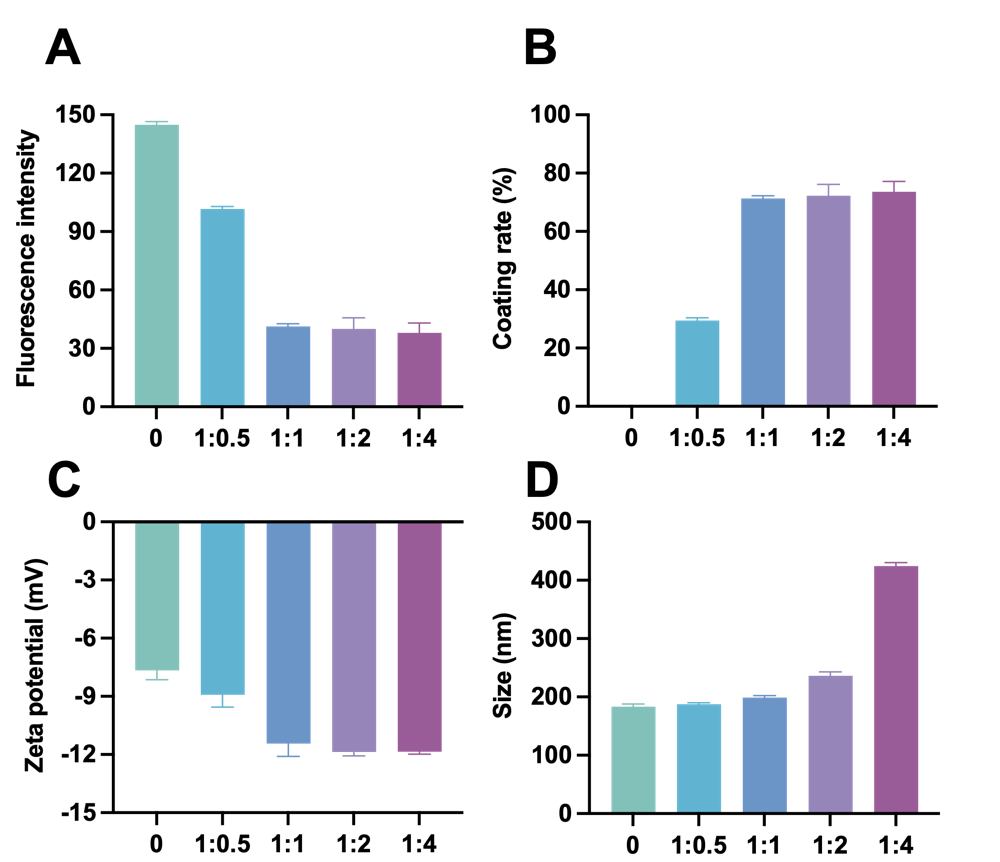


Figure S2. Study on the encapsulation efficiency of RBC Membrane. Fluorescence intensity (A), encapsulation efficiency(B), Zeta potential (C) and particle size (D) of W/HPDA/L@RBC-BOR nanoparticles extruded at different mass ratios of W/HPDA/L nanoparticles to RBC membranes. Data are presented as mean ± SD (n=3).


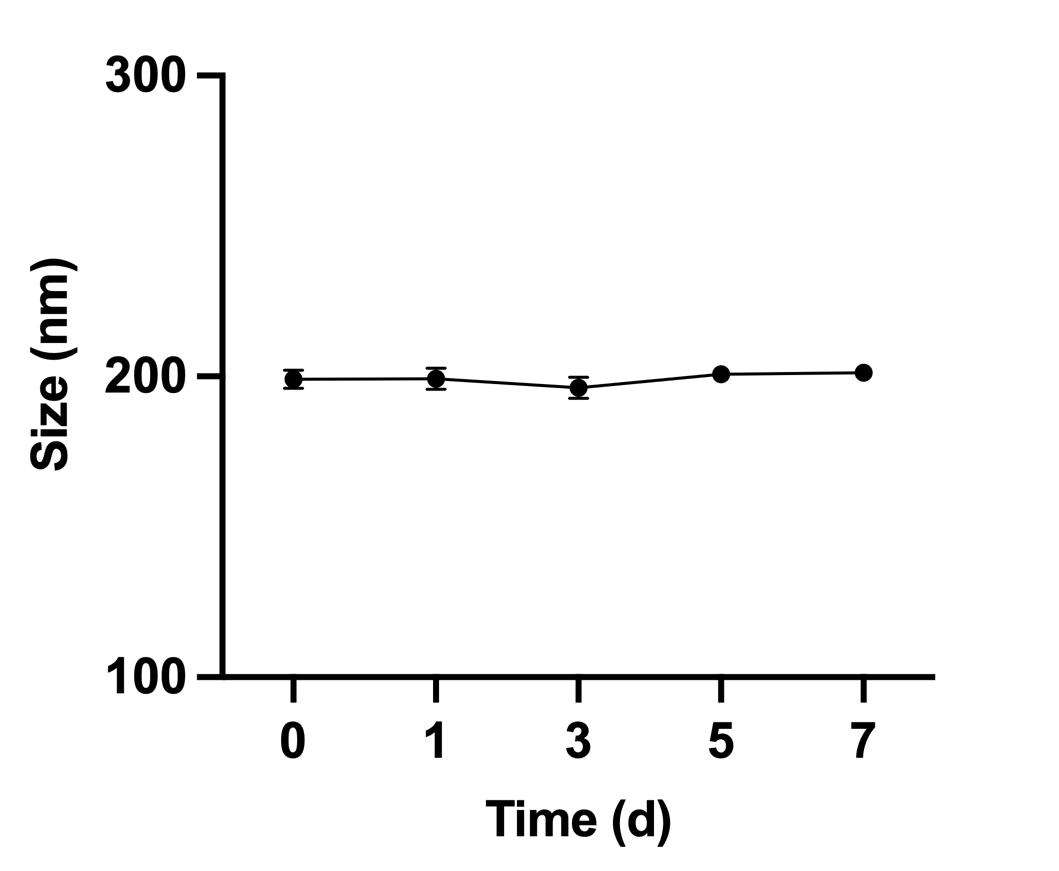


Figure S3. Particle size changes of W/HPDA/L@RBC-BOR nanoparticles at 4°C (n=3).


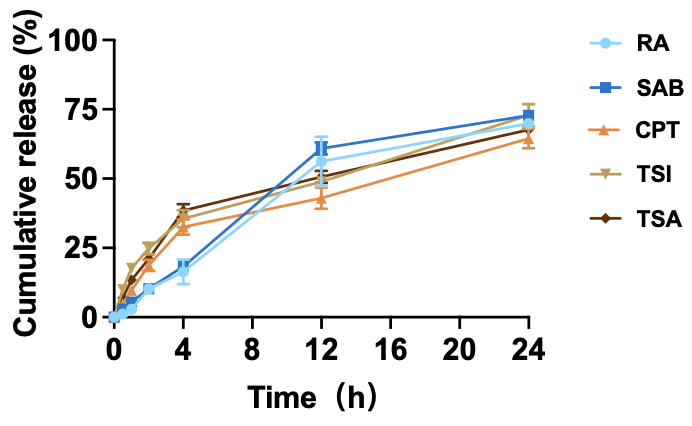


Figure S4. *In vitro* release profiles of five compounds from W/HPDA/L@RBC-BOR in 1% SDS (pH 6.5-6.8) (n=3).


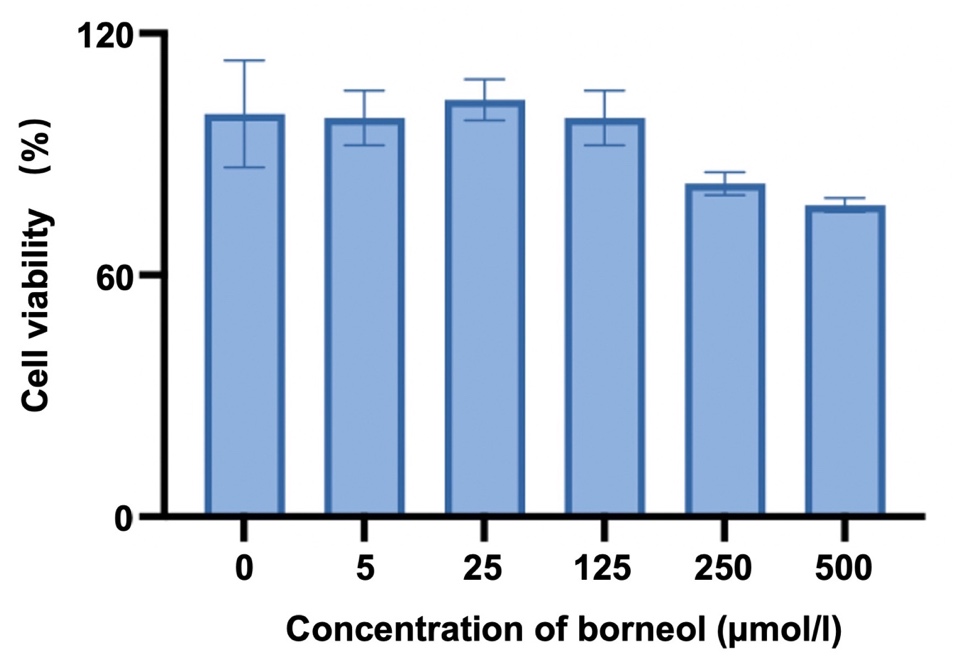


Figure S5. Cell viability of bEnd.3 cells incubated with different doses of borneol (n=5).


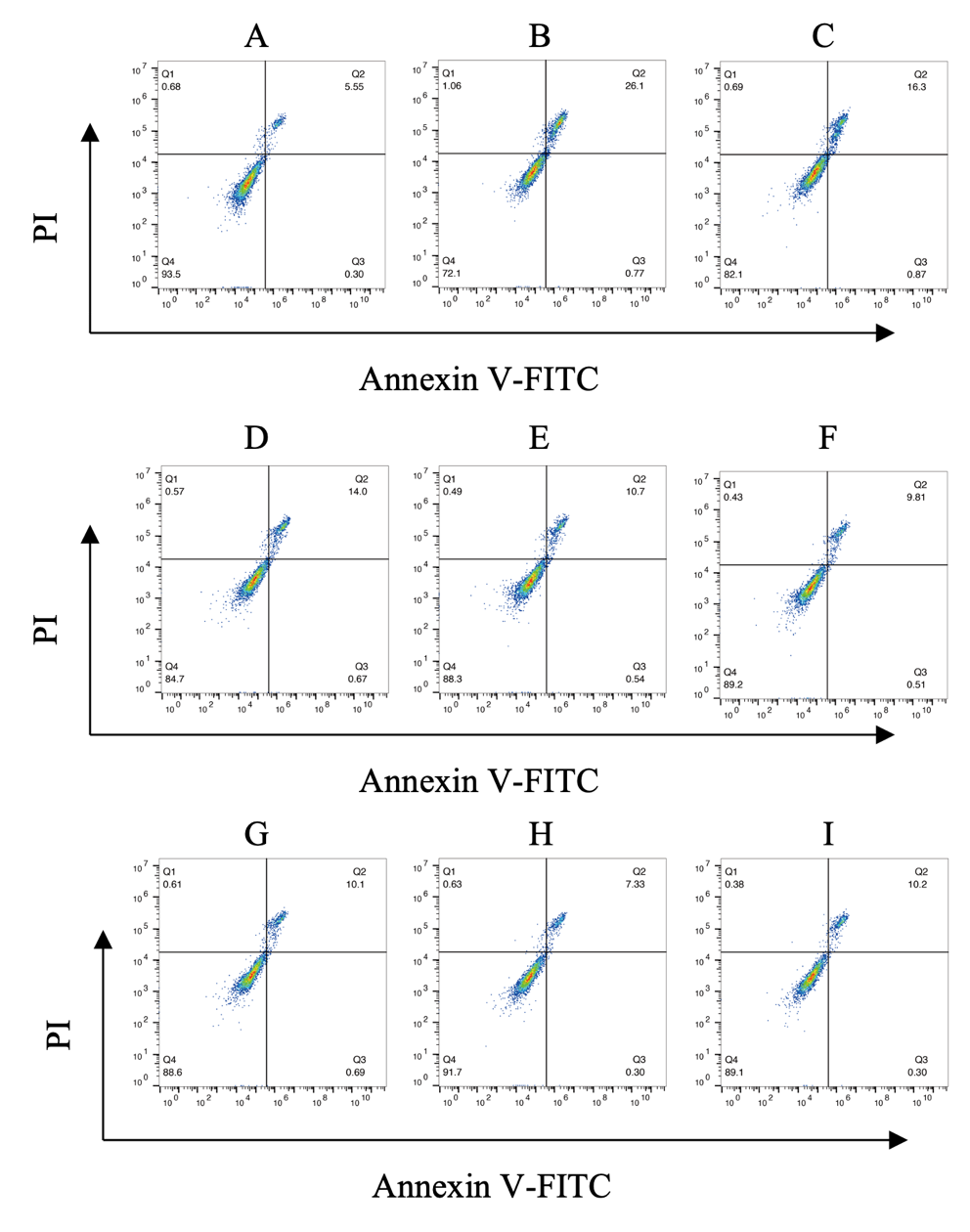


Figure S6. Representative images of cell apoptosis analyzed with flow cytometry, where HT22 cells were treated with nine group: control (A), model (B), free drug (C), W/HPDA/L (D), W/HPDA/L@RBC (E), W/HPDA@RBC-BOR (F), HPDA/L@RBC-BOR (G), W/HPDA/L@RBC-BOR (H), HPDA/WL@RBC-BOR (I). Data are presented as mean ± SD (n = 3).


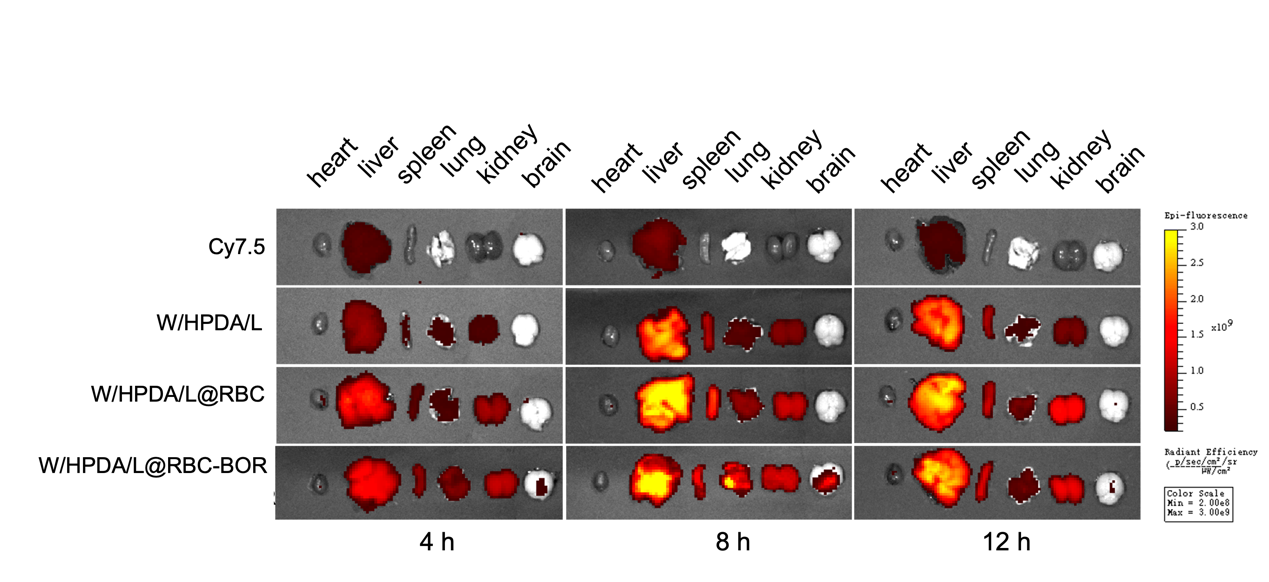


Figure S7. Representative ex vivo fluorescence imaging of the brain, heart, lung, liver, kidney, and spleen of mice at 4, 8 and 12 h after administration of cy7.5, W/HPDA/L, W/HPDA/L@RBC and W/HPDA/L@RBC-BOR.


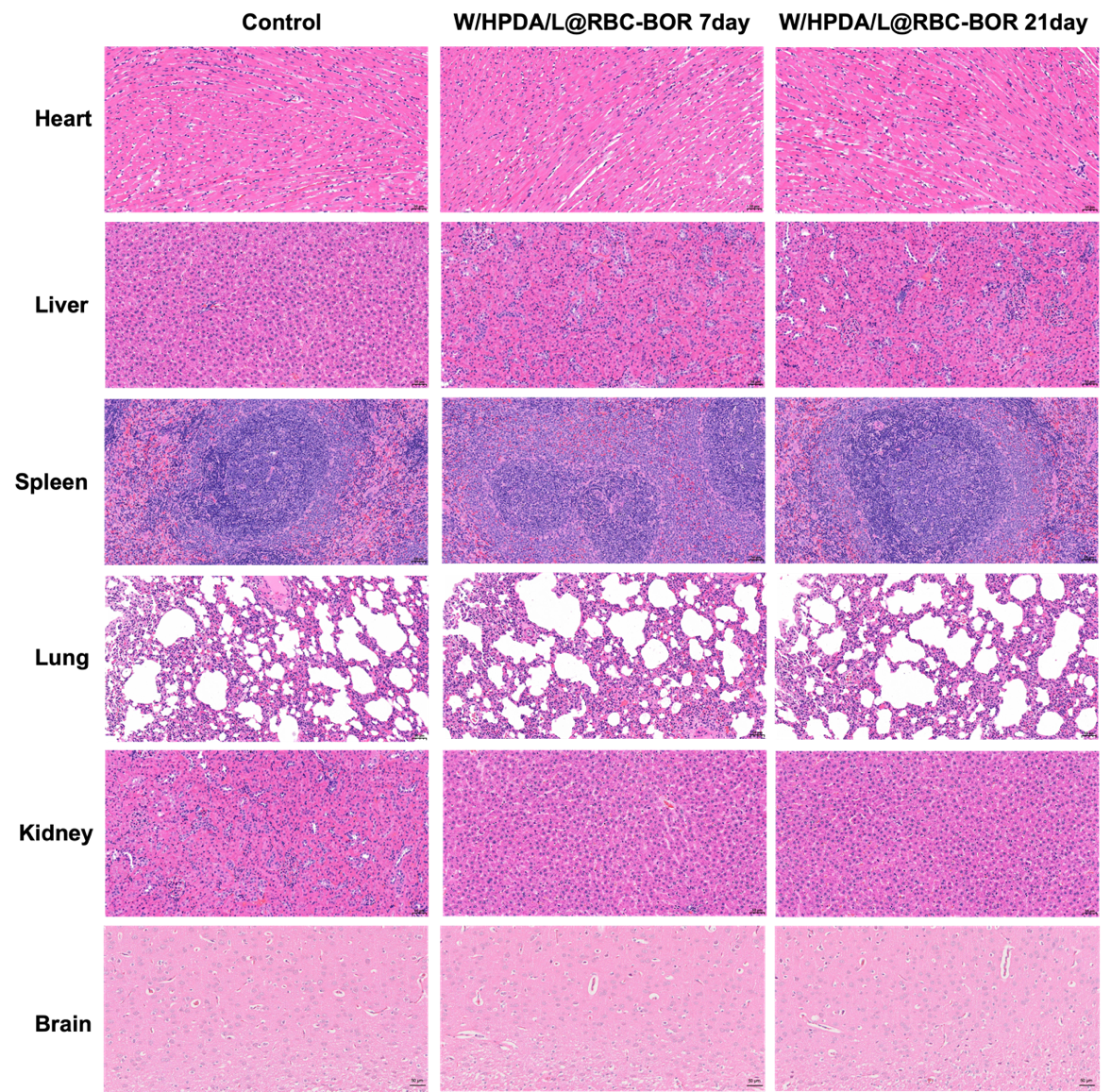


Figure S8. H&E staining of main organs at 7 and 21 days after W/HPDA/L@RBC-BOR treatment. Scale bars, 50 μm.


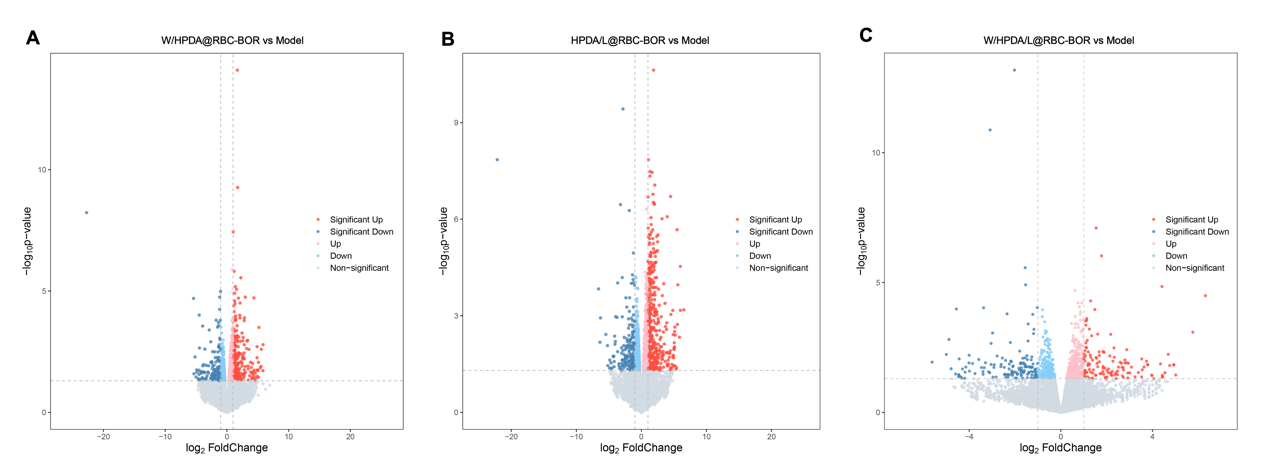


Figure S9. Volcano plots for all differentially expressed genes following treatment with W/HPDA@RBC-BOR, HPDA/L@RBC-BOR and W/HPDA/L@RBC-BOR. Red indicates upregulated, while green indicates downregulated.

Table S1. RT-qPCR genes and primer sequences used for brain tissue samples.

| Gene | Primer | Sequence (5’—3’) |
| --- | --- | --- |
| *GAPDH* | forward | CATGGCCTTCCGTGTTCCTA |
|  | reverse | CCTGCTTCACCACCTTCTTGAT |
| *Brca1* | forward | GAGCTGGAGATGAAGGCAAG |
|  | reverse | CTGCATCCAGTCTGTCCTCA |
| *Cxcl1* | forward | GCACCCAAACCGAAGTCATA |
|  | reverse | GGGGACACCCTTTAGCATCT |
| *Cxcl2* | forward | TGGTCCTCGTCTTCCTGCTCTG |
|  | reverse | CGTTCTGGCGTTCACAGGTCTC |
| *Col1a1* | forward | TGTTGGTCCTGCTGGCAAGAATG |
|  | reverse | GTCACCTTGTTCGCCTGTCTCAC |
| *Gadd45b* | forward | GAAGAAGAGGAGGATGATATTG |
|  | reverse | CAGTTCGTGACCAGGAG |
| *Lif* | forward | ATGCCCTCTTTATTTCCTATTACAC |
|  | reverse | CATGACGTCTGTAGTCGCATTGAGT |
| *Fgf2* | forward | ACCCGGCCACTTCAAGG |
|  | reverse | GATGCGCAGGAAGAAGCC |
| *Tlr2* | forward | CAGCGAAAATCTGATGGTTGAAG |
|  | reverse | GATGTCAAGGGCTGTCAGGTTT |
| *Birc3* | forward | AGCTACCTCTCAGCCTACTTT |
|  | reverse | CCACTGTTTTCTGTACCCGGA |
| *Gadd45g* | forward | GTTGATCCAGGCGTTCT |
|  | reverse | GGTCCTTCCATGTGTCTT |
| *Fos* | forward | GGAGGACCTTATCTGTGCGT |
|  | reverse | TGCGGTTGCTTTTGATTTTT |
| *Junb* | forward | TCTTTCTCTTCACGACTACA |
|  | reverse | CTAGCTTCAGAGATGCG |

Table S2. Pharmacokinetic Parameters of RA after free drug, W/HPDA/L, W/HPDA/L@RBC and W/HPDA/L@RBC-BOR injection (n=3).

| Parameter | Free Drug | W/HPDA/L | W/HPDA/L@RBC | W/HPDA/L  @RBC-BOR |
| --- | --- | --- | --- | --- |
| AUC_(0-t)_  (h·mg/l) | 3.332±0.104^&&&##^ | 4.508±0.166^&&#^ | 10.001±1.179 | 11.9366±0.203 |
| AUC_(0-∞)_  (h·mg/l) | 3.343±0.102^&&##^ | 4.703±0.233^&&#^ | 13.019±1.260 | 16.371±0.122 |
| MRT_(0-∞)_ (h) | 0.097±0.036^&&&###^ | 0.305±0.041^&&&###^ | 0.705±0.083 | 0.719±0.011 |
| t_1/2_ (h) | 0.135±0.043^&&&###^ | 0.397±0.099^&&##^ | 0.957±0.050 | 1.081±0.047 |
| C_max_ (mg/l) | 14.861±0.799 | 16.062±0.985 | 10.944±2.046 | 12.913±0.706 |

Table S3. Pharmacokinetic Parameters of SAB after free drug, W/HPDA/L, W/HPDA/L@RBC and W/HPDA/L@RBC-BOR injection.

| Parameter | Free Drug | W/HPDA/L | W/HPDA/L@RBC | W/HPDA/L  @RBC-BOR |
| --- | --- | --- | --- | --- |
| AUC_(0-t)_ (h·mg/l) | 3.968±1.905^&&&###^ | 6.958±0.930^&&###^ | 11.843±0.112 | 16.675±0.149 |
| AUC_(0-∞)_ (h·mg/l) | 3.986±1.613^&&&###^ | 7.719±0.670^&&###^ | 14.801±1.231 | 20.716±2.137 |
| MRT_(0-∞)_ (h) | 0.093±0.034^&&##^ | 0.392±0.032^&#^ | 0.609±0.037 | 0.635±0.052 |
| t_1/2_ (h) | 0.116±0.007^&&###^ | 0.521±0.034^#^ | 0.921±0.172 | 0.985±0.281 |
| C_max_ (mg/l) | 23.350±4.147 | 27.897±2.234 | 22.815±1.278 | 20.836±0.881 |

Table S4. Pharmacokinetic Parameters of CPT after free drug, W/HPDA/L, W/HPDA/L@RBC and W/HPDA/L@RBC-BOR injection.

| Parameter | Free Drug | W/HPDA/L | W/HPDA/L@RBC | W/HPDA/L  @RBC-BOR |
| --- | --- | --- | --- | --- |
| AUC_(0-t)_  (h·mg/l) | 3.685±0.702^&&&###^ | 5.104±0.654^&&&###^ | 9.098±0.487 | 10.017±1.0515 |
| AUC_(0-∞)_  (h·mg/l) | 3.767±0.725^&&&###^ | 5.636±1.207^&&&###^ | 13.031±3.292 | 16.471±2.607 |
| MRT_(0-∞)_ (h) | 0.284±0.151^&&##^ | 0.442±0.088 | 0.801±0.053 | 0.807±0.051 |
| t_1/2_ (h) | 0.346±0.132^&##^ | 0.559±0.371 | 1.161±0.509 | 1.485±0.451 |
| C_max_ (mg/l) | 12.276±1.748 | 13.416±0.989 | 8.943±0.653 | 8.889±0.553 |

Table S5. Pharmacokinetic Parameters of TSI after free drug, W/HPDA/L, W/HPDA/L@RBC and W/HPDA/L@RBC-BOR injection.

| Parameter | Free Drug | W/HPDA/L | W/HPDA/L@RBC | W/HPDA/L  @RBC-BOR |
| --- | --- | --- | --- | --- |
| AUC_(0-t)_  (h·mg/l) | 2.216±0.448^&&&###^ | 3.496±0.737^&&&###^ | 6.793±0.726 | 8.366±0.327 |
| AUC_(0-∞)_  (h·mg/l) | 2.222±0.456^&&&###^ | 3.717±0.664^&&###^ | 8.399±1.846 | 14.082±1.448 |
| MRT_(0-∞)_ (h) | 0.198±0.063^&&&###^ | 0.467±0.024^&##^ | 0.754±0.056 | 0.841±0.085 |
| t_1/2_(h) | 0.314±0.077^&&###^ | 0.529±0.125^&##^ | 0.813±0.237 | 1.518±0.646 |
| C_max_(mg/l) | 7.498±1.055 | 7.861±0.243 | 6.296±0.114 | 6.154±0.266 |

Table S6. Pharmacokinetic Parameters of TSA after free drug, W/HPDA/L, W/HPDA/L@RBC and W/HPDA/L@RBC-BOR injection.

| Parameter | Free Drug | W/HPDA/L | W/HPDA/L@RBC | W/HPDA/L  @RBC-BOR |
| --- | --- | --- | --- | --- |
| AUC_(0-t)_  (h·mg/l) | 2.286±1.623^&&&###^ | 4.613±0.227^&&&###^ | 11.435±1.159 | 13.504±0.621 |
| AUC_(0-∞)_  (h·mg/l) | 2.292±1.527^&&&###^ | 4.867±0.219^&&&###^ | 15.642±2.508 | 21.131±1.772 |
| MRT_(0-∞)_ (h) | 0.162±0.031^&&&###^ | 0.446±0.071^&&&###^ | 0.756±0.097 | 0.781±0.089 |
| t_1/2_(h) | 0.326±0.092^&&&###^ | 0.611±0.045 | 1.012±0.465 | 1.355±0.285 |
| C_max_(mg/l) | 15.728±0.473 | 16.334±2.874 | 12.933±2.761 | 12.698±0.803 |

Data are presented as mean ± SD (n=3). ^&^*P*<0.05, ^&&^*P*<0.01, ^&&&^*P*<0.001 *vs*. W/HPDA/L@RBC group; ^#^*P*<0.05, ^##^*P*<0.01, ^###^*P*<0.001 *vs*. W/HPDA/L@RBC-BOR group.
